# Supplementary material for: Structural and functional characterization of the intracellular filament-forming nitrite oxidoreductase multiprotein complex
Source: Nat Microbiol. 2021 Jul 15;6(9):1129–39. doi: 10.1038/s41564-021-00934-8 (PMC8387239; doi:10.1038/s41564-021-00934-8)
Supplement: Supplementary file 1 — Supplementary text, Tables 1–3 and references. [file 41564_2021_934_MOESM1_ESM.pdf]

nature portfolio

https://doi.org/10.1038/s41564-021-00934-8

Supplementary information

Structural and functional characterization of the intracellular filament-forming nitrite oxidoreductase multiprotein complex

In the format provided by the authors and unedited

Supplementary Information for

Structural and functional characterization of the intracellular filament-forming Nitrite Oxidoreductase multiprotein complex

Authors: Tadeo Moreno Chicano†[1], Lea Dietrich†[2], Naomi M. de Almeida†[3], Mohd. Akram[1], Elisabeth Hartmann[1], Franziska Leidreiter[1], Daniel Leopoldus[1], Melanie Mueller[1], Ricardo Sánchez[2,5], Guylaine Nuijten[3], Joachim Reimann[3], Kerstin-Anikó Seifert[1], Ilme Schlichting[1], Laura van Niftrik[3], Mike S.M. Jetten[3], Andreas Dietl*[1], Boran Kartal*[4,6], Kristian Parey*[2,7] and Thomas R.M. Barends*[1]

KsNXR-ABC contains a high-potential haem

The UV/Vis spectrum of KsNXR-ABC features a sharp peak at 425 nm indicative of a haem, and α- and β- absorptions at 527 nm and 561 nm (Extended Data Figure 2) that are typical for a *b*-type haem in its reduced state. The addition of the strong reductant sodium dithionite (E_0' ~ -0.66 V at pH 7) did not result in a shift of this peak, suggesting that the haem is completely reduced in the as-isolated state, even though the protein was purified under aerobic conditions. The addition of the oxidant potassium ferricyanide (E_0' ~ 0.43 V at pH 7) in the presence of oxygen resulted in a broadening and shift of the Soret band, with the maximum moving to 415 nm, and a decrease of the alpha and beta bands. The UV-Vis spectra suggest reduction of the haem when ferricyanide-oxidized KsNXR-ABC was titrated with nitrite, ascorbate or dithionite. Addition of sodium nitrate to the reduced enzyme did not result in oxidation of the haem.

Crystal structure of KsNXR-ABC

KsNXR-ABC was crystallized with eight NXR-A/NXR-B/NXR-C heterotrimers in the asymmetric unit, which form a short stretch of a double helix, with its two strands composed of four heterotrimers each. Each strand contains a head-to-tail dimer of heterotrimers, held together by predominantly polar interactions, capped on either side by a further heterotrimer. Interestingly, this head-to-tail dimeric configuration is also observed in the tubules, with NXR-T subunits taking the positions of the "capping heterotrimers" (Extended Data Figure 3a). The present resolution of the EM results does not, however, allow us to ascertain whether the interactions in the head-to-tail dimers are identical between the crystal- and tubule structures. The NXR A, B and C subunits form a closely intertwined heterotrimer; the surface area buried upon complex formation as determined by PISA[1] is >25,000 Å. The side chains of Glu1088A, Asp611A and the OH group of Tyr528A form the entrance to the tunnel leading to the active site. The residues lining the tunnel are predominantly polar, but include several aromatic residues. At the bottom, close to the active site, the tunnel's surface is positively charged as determined using APBS[2].

In the active site itself (Figure 2A), the electron density maps clearly show that in both pterin cofactors coordinating the molybdenum, the pyran rings are in the 'open' state. Extended Data Figure 3b shows a simulated annealing omit map, locally averaged using custom-written code[3]

over 6 monomers of the proximal pterin's pyran ring. To verify that this opening of the pyran rings is not due to radiation damage, we divided the original dataset into two parts; one containing the first half of the images collected, and one containing the second half. These two parts should differ in absorbed radiation dose, with any radiation damage being more pronounced in the latter half of the data. However, difference electron density maps calculated between the two parts of the dataset did not show any features that suggest the pyran ring opening of either pterin molecule is due to radiation damage.

Crystal structures in which only the distal pterin adopts a pyran-open configuration have been reported for NAR[4] and EBDH[5], and in another Nar structure it could not be excluded[6]. In the KsNXR crystal structure, however, both distal and proximal pterin molecules are in the pyran-open state. In KsNXR, the hydroxyl group of the open pyran ring of the distal pterin is hydrogen bonded to His1020A, similar to the situation in EBDH[5] where this OH interacts with the homologous His855. The proximal pyran OH group contacts the Asn312A Oδ1 atom, but does not appear to form a hydrogen bond given the mutual orientation. In between the pterins, Lys917A is hydrogen bonded to the N15 atom of the pyrazine ring of the distal pterin only. In both the EBDH and *E. coli* Nar structures, this residue is a histidine that interacts with the N15 atoms of both distal- and proximal pterins. The effect of pyran ring opening has been an intensely studied topic[7-13]. Interestingly, opening and closing this ring involves redox chemistry, with ring opening corresponding to a two-electron oxidation[13], and it was suggested early on that ring opening/closing could play a part in catalysis[14]. Later, it was recognized that respiratory nitrate reductases are often purified in a partially inactive state that must be activated by a reductive process, and it was proposed that this activation involves closure of a molybdopterin pyran ring[15]. Possibly, pyran ring opening serves a regulatory function in NXRs, too.

Identification of Kustd1705 as the connecting density

To identify the constituents of the connecting density, we isolated intact NXR tubules from *K. stuttgartiensis* by size exclusion chromatography and density gradient centrifugation, separated the subunits by SDS-PAGE and performed peptide mass fingerprinting (see Extended Data Figure 5). In addition to the A-, B- and C- subunits of KsNXR, this revealed the presence of the monohaem c-type cytochrome Kustd1705, which is encoded by the nxr gene cluster in the tubule preparation. We expressed Kustd1705 in *Shewanella oneidensis*, and performed *in vitro* reconstitution assays with KsNXR-ABC and Kustc1705 using a range of conditions, testing salt concentrations and additives (nitrite-, nitrate-, calcium- and magnesium ions), screening for tubule formation by negative stain EM. When none of these experiments proved successful, we studied the stability of isolated tubules in various conditions. This revealed that the presence of the detergent dodecyl maltoside rapidly and completely destroys isolated tubules. As this detergent was used in the initial steps of the purification of KsNXR-ABC, it appeared possible that some remaining dodecyl maltoside inhibited tubule formation in our reconstitution experiments. We therefore treated KsNXR-ABC with BioBeads® (Bio-Rad, Feldkirchen, Germany) to remove residual detergent and repeated the reconstitution screens. This revealed that the addition of Kustd1705 (without His-tag) resulted in tubule reconstitution. Thus, we propose to name Kustd1705 "NXR-T" for tubule-inducing NXR subunit.

Characterization and structure determination of Kustd1705 (KsNXR-T) and its Brocadia fulgida homolog Broful_01488

Heterologously expressed Kustd1705 and Broful_01488 were both red proteins in solution, indicating haem incorporation as expected. UV/Vis spectroscopy of both proteins indeed showed strong Soret absorptions which displayed a red shift on reduction (Extended Data Figure 7): the Soret maximum shifted from 410 nm to 419 on reduction of Kustd1705, and from 415 to 417.5 nm upon reduction of Broful_01488. Possibly, as-isolated Broful_01488 is not fully reduced. The UV/Vis spectra were not affected by 10 mM of either nitrite, nitrate or imidazole (not shown).

As KsNXR-T did not crystallize, we reconstituted tubules an analyzed them by helical reconstruction cryoelectron microscopy. This resulted in a map which has an overall resolution of 5.8 Å, clearly showing a four-helix bundle fold for the NXR-T monomer (Extended Data Figure 6d). The position of the haem groups is clear from the maps' highest peak. The resolution of the cryo-EM map in the region of the KsNXR-ABC trimers is considerably lower (Extended Data Figure 6c). A homology model of KsNXR-T was built and fitted into the density, assuming that the haem group is covalently bound to the canonical CxxCH haem binding motif close to the C-terminus (Extended Data Figure 7), i.e. with the two cysteines bound to the haem vinyl groups and the histidine coordinating the haem iron on the proximal side. Due to the limited resolution, details such as the distal haem coordination cannot be resolved, however, the model suggests that the N-terminal helix lies on the distal side of the haem. This helix contains a conserved Met residue, which could be the distal haem ligand (Extended Data Figure 7h); His/Met ligation would be consistent with the observed redox potential and UV/Vis spectrum.

The crystals of the *Brocadia fulgida* homolog Broful_01488 diffracted poorly and the resulting 4.0-4.5 Å resolution electron density map also does not show sufficient detail to decide on the haem binding mode. However, the Broful_01488 model does confirm the four-helix bundle fold and dimer structure of NXR-T.

Moreover, both Kustd1705 (KsNXR-T) and Broful_01488 are dimers in solution as shown by sedimentation velocity ultracentrifugation (Extended Data Figure 7d). This method records the change in concentration profile of the protein under study as it is being sedimented. The resulting data can then be translated into a continuous distribution of species with a certain sedimentation coefficient. This value can then be extrapolated to a sedimentation coefficient $S_{w,20}$ that would have been observed in water at 20° C. This value can now be compared to the predicted maximum values of the sedimentation coefficient for different oligomeric states of a protein, that can be calculated from their molecular masses, as described in detail by Erickson[16]. Briefly, the highest possible sedimentation coefficient S_{max}, which is the theoretical sedimentation coefficient for a perfect sphere, is calculated from the molecular mass M (in Da) using the formula $S_{max} = 0.00361M^{2/3}$ [16]. The comparison of the observed sedimentation coefficient with the calculated S_{max} for a certain oligomeric state is done by calculating the ratio $S_{max}/S_{w,20}$. For globular proteins, this ratio is typically around 1.2-1.3, and is larger for shapes that are more elongated (for instance, for TNfn1-5, which is ~5 times longer than it is broad, it is 1.65[16]). A value lower than 1.0 would mean that the protein is more spherical than a perfect sphere, which is impossible. Thus, one calculates the value of $S_{max}/S_{w,20}$ for different possible oligomeric states, and the oligomer with the most reasonable value of $S_{max}/S_{w,20}$ given what is known about the protein otherwise, is then the most likely oligomeric state.

In the present case, for Kustd1705, a sedimentation coefficient of 3.09 S was determined, which extrapolates to $S_{w,20}$=3.10 S in water at 20° C. Given the calculated molecular mass of the

monomer of 17,300.65 Da, values of S_{max} of 2.41, 3.83 and 5.02 S can be calculated for a monomer, dimer and trimer, respectively, using the formula $S_{max} = 0.00361M^{2/3}$[16]. Of these values, only the value for the dimer (3.83 S) results in a realistic ratio $S_{max}/S_{w,20}$=1.24 for a more or less globular protein (the other two values result in values for $S_{max}/S_{w,20}$ of 0.77, which is unphysically small, and 1.62, which would indicate an extremely elongated shape, which we have no reason to expect). The results therefore suggest a dimeric state in solution for Kustd1705. Moreover, calculations of the sedimentation coefficient from the dimeric structural model using HYDROPRO[17] resulted in a predicted value of 3.1 S, which fits the observed sedimentation coefficient excellently.

Similarly, a sedimentation coefficient of 2.98 S was measured for Broful_01488, corresponding to $S_{w,20}$=2.99 S. Given that the calculated masses for the monomeric, dimeric and trimeric states are 16,593.97, 33,187.94 and 49,781.91 Da, respectively, again only the dimeric state would result in a realistic ratio $S_{max}/S_{w,20}$=1.25. In addition, we also calculated the predicted sedimentation coefficient for the dimer observed in the crystal structure, which resulted in a value of 3.2 S which is also in line with the observed sedimentation coefficient. Thus, both proteins appear to behave as dimers in solution.

Supplementary Table S1.

Data collection, phasing and refinement statistics.

	KsNXR-ABC	*Brocadia fulgida* NXR-T (Broful_01488)
**Data collection**		
Space group	$P2_12_12_1$	$C222_1$
Cell dimensions		
a, *b*, *c* (Å)	177.6, 206.5, 527.1	78.5, 88.4, 95.7
α, β, γ (°)	90, 90, 90	90, 90, 90
Wavelength	1.73450	1.73728
Resolution (Å)	206 - 3.0 (3.1 - 3.0)*	40 - 4.5 (4.6 - 4.5)
R_{pim}	0.085 (0.570)	0.077 (1.34)
$I / \sigma I$	9.6 (1.4)	7.9 (1.9)
Completeness (%)	96.7 (62.1)	100.0 (91.3)
Redundancy	26.3 (20.6)	7.0 (6.7)
CC_{ano}	0.14 (0.0)	0.38 (0.04)
**Refinement**		
Resolution (Å)	106 - 3.0	---no full model refined---
No. reflections	377,164	
R_{work} / R_{free}	0.2230/0.2550	
No. atoms		
Protein	113,710	
Ligand/ion	1416	
Water	799	
B-factors		
Protein	47.4	
Ligand/ion	48.8	
Water	30.6	
R.m.s deviations		
Bond lengths (Å)	0.006	
Bond angles (°)	1.068	

*Values for the highest resolution shell are given in parentheses.

Supplementary Table S2. Subtomogram averaging statistics

	Subtomogram averaging (EMD-11860)
**Data collection**	
Microscope	FEI Titan Krios
Camera	Gatan K2 Summit
Voltage (kV)	300
Nominal magnification	42 000×
Calibrated pixel size (Å)	3.585 Å
Electron dose per tomogram ($e^{-}/Å^{2}$)	~100.0
Defocus range (µm)	3.5 – 4.5
**Image processing**	
Motion correction software	*MotionCor2*
Tomogram reconstruction software	*IMOD*
Subtomogram averaging software	*Dynamo*
Number of tomograms	18
Initial particle images (no.)	7109
Final particle images (no.)	5126
Final resolution (Å)	22 Å

Supplementary Table S3. Cryo-EM helical reconstruction data statistics

	Helical reconstruction (EMDB-11861)
**Data collection and processing**	
Magnification	105,000×
Voltage (kV)	300
Electron exposure (e–/$Å^2$)	50.0
Defocus range (μm)	2.0-3.0
Pixel size (Å)	0.837
Symmetry imposed	-none-
Initial particle images (no.)	9,275
Final particle images (no.)	5,379
Map resolution (Å)	5.8
FSC threshold	0.143
Map resolution range (Å)	355 – 5.8
**Helical parameters***	
twist	121°
rise	108 Å

* determined using Chimera[18]

Supplementary References

1 Krissinel, E. & Henrick, K. Inference of macromolecular assemblies from crystalline state. *J Mol Biol* **372**, 774-797 (2007).

2 Jurrus, E. *et al.* Improvements to the APBS biomolecular solvation software suite. *Prot. Sci.* **27**, 112-128, doi:10.1002/pro.3280 (2018).

3 Nass, K. *et al.* Structural dynamics in proteins induced by and probed with X-ray free-electron laser pulses. *Nat. Comm.* **11**, doi:10.1038/s41467-020-15610-4 (2020).

4 Bertero, M. G. *et al.* Insights into the respiratory electron transfer pathway from the structure of nitrate reductase A. *Nat. Struct. Biol.* **10**, 681–687, doi:10.1038/nsb969 (2003).

5 Kloer, D. P., Hagel, C., Heider, J. & Schulz, G. E. Crystal structure of ethylbenzene dehydrogenase from *Aromatoleum aromaticum*. *Structure* **14**, 1377-1388, doi:10.1016/j.str.2006.07.001 (2006).

6 Jormakka, M., Richardson, D., Byrne, B. & Iwata, S. Architecture of NarGH reveals a structural classification of Mo-bisMGD enzymes. *Struct.* **12**, 95-104, doi:10.1016/j.str.2003.11.020 (2004).

7 Gisewhite, D. R. *et al.* Implications of Pyran Cyclization and Pterin Conformation on Oxidized Forms of the Molybdenum Cofactor. *Journal of the American Chemical Society* **140**, 12808-12818, doi:10.1021/jacs.8b05777 (2018).

8 Nguyen, N. K. N., Williams, B., Gisewhite, D. & Burgmayer, S. N. Molybdenum pyranopterin dithiolene complexes: Synthetic model and investigation in the role of pterin reduction in the molybdenum cofactor. *Abstr. Pap. Am. Chem. Soc.* **253** (2017).

9 Nagelski, A., Gisewhite, D., Williams, B. & Burgmayer, S. N. Probing pterin reduction dynamics in synthetic molybdenum cofactor models. *Abstr. Pap. Am. Chem. Soc.* **253** (2017).

10 Burgmayer, S. J. N., Williams, B. R. & Basu, P. Pterin-inspired model compounds of molybdenum enzymes. *Molybd. Tungst Enz.: Bioinorg. Chem.* **6**, 8-67 (2017).

11 Zhu, S. J., Williams, B. & Burgmayer, S. N. Pterin reduction in chemical models of the molybdenum cofactor. *Abstr. Pap. Am. Chem. Soc.* **251** (2016).

12 Burgmayer, S. N., Gisewhite, D., Williams, B. & Zhu, S. J. Investigating the role of the pterin dithiolene ligand in the molybdenum cofactor. *Abstr. Pap. Am. Chem. Soc.* **252** (2016).

13 Basu, P. & Burgmayer, S. J. N. Pterin chemistry and its relationship to the molybdenum cofactor. *Coord. Chem. Rev.* **255**, 1016-1038, doi:10.1016/j.ccr.2011.02.010 (2011).

14 Enemark, J. H. & Garner, C. D. The coordination chemistry and function of the molybdenum centres of the oxomolybdoenzymes. *J. Biol. Inorg. Chem.* **2**, 817-822, doi:10.1007/s007750050201 (1997).

15 Jacques, J. G. J. *et al.* Reductive activation in periplasmic nitrate reductase involves chemical modifications of the Mo-cofactor beyond the first coordination sphere of the metal ion. *Biochim. Biophys. Act.-Bioenerg.* **1837**, 277-286, doi:10.1016/j.bbabio.2013.10.013 (2014).

16 Erickson, H. P. Size and shape of protein molecules at the nanometer level determined by sedimentation, gel filtration, and electron microscopy. *Biol. Proced. Online* **11**, 32-51, doi:10.1007/s12575-009-9008-x (2009).

17 Ortega, A., Amoros, D. & de la Torre, J. G. Prediction of Hydrodynamic and Other Solution Properties of Rigid Proteins from Atomic- and Residue-Level Models. *Biophys J* **101**, 892-898, doi:10.1016/j.bpj.2011.06.046 (2011).

18 Goddard, T. D. *et al.* UCSF ChimeraX: meeting modern challenges in visualization and analysis. *Prot. Sci.* **27**, 14-25, doi:10.1002/pro.3235 (2018).
