# Supplementary material for: Structural and functional characterization of the intracellular filament-forming nitrite oxidoreductase multiprotein complex
Source: Nat Microbiol. 2021 Jul 15;6(9):1129–39. doi: 10.1038/s41564-021-00934-8 (PMC8387239; doi:10.1038/s41564-021-00934-8)
Supplement: Supplementary file 2 — Reporting Summary [file 41564_2021_934_MOESM2_ESM.pdf]

nature research

Corresponding author(s): Andreas Dietl
Boran Kartal
Kristian Parey
Thomas Barends

Last updated by author(s): 27/5/21

Reporting Summary

Nature Research wishes to improve the reproducibility of the work that we publish. This form provides structure for consistency and transparency in reporting. For further information on Nature Research policies, see our Editorial Policies and the Editorial Policy Checklist.

Statistics

For all statistical analyses, confirm that the following items are present in the figure legend, table legend, main text, or Methods section.

n/a	Confirmed	
☒	☐	The exact sample size (*n*) for each experimental group/condition, given as a discrete number and unit of measurement
☐	☒	A statement on whether measurements were taken from distinct samples or whether the same sample was measured repeatedly
☒	☐	The statistical test(s) used AND whether they are one- or two-sided<br>*Only common tests should be described solely by name; describe more complex techniques in the Methods section.*
☒	☐	A description of all covariates tested
☒	☐	A description of any assumptions or corrections, such as tests of normality and adjustment for multiple comparisons
☒	☐	A full description of the statistical parameters including central tendency (e.g. means) or other basic estimates (e.g. regression coefficient) AND variation (e.g. standard deviation) or associated estimates of uncertainty (e.g. confidence intervals)
☒	☐	For null hypothesis testing, the test statistic (e.g. *F*, *t*, *r*) with confidence intervals, effect sizes, degrees of freedom and *P* value noted<br>*Give P values as exact values whenever suitable.*
☒	☐	For Bayesian analysis, information on the choice of priors and Markov chain Monte Carlo settings
☒	☐	For hierarchical and complex designs, identification of the appropriate level for tests and full reporting of outcomes
☒	☐	Estimates of effect sizes (e.g. Cohen's *d*, Pearson's *r*), indicating how they were calculated

Our web collection on statistics for biologists contains articles on many of the points above.

Software and code

Policy information about availability of computer code

Data collection	Crystallographic data collection software: XDS (version 10.1.2014) | CryoEM: MotionCor 1.2.6, IMOD 4.10.9, SerialEM 3.6.22
Data analysis	Crystallographic data analysis software: PHENIX (version 1.14rc1_3177), CCP4 7.1.003, PHASER 2.8.3. EM: NovaCTF 4f134c7, Dynamo 1.1.478, EMAN 2.31, CTFFIND 4.1.13, RELION 3.1. Local density averaging as described in the referenced paper. Figures prepared with Chimera 1.14, Pymol 2.4.1,Origin 9.6.5,ESPript 3.0, GraFit 7.0.0

For manuscripts utilizing custom algorithms or software that are central to the research but not yet described in published literature, software must be made available to editors and reviewers. We strongly encourage code deposition in a community repository (e.g. GitHub). See the Nature Research guidelines for submitting code & software for further information.

Data

Policy information about availability of data

All manuscripts must include a data availability statement. This statement should provide the following information, where applicable:

- Accession codes, unique identifiers, or web links for publicly available datasets
- A list of figures that have associated raw data
- A description of any restrictions on data availability

The NXR-ABC crystal structure and structure factor amplitudes were deposited in the PDB under accession code 7B04. EM maps are available from the EMDB under accession codes EMD-11860 and EMD-11861. These repositories are publicly accessible. Raw data are associated with this paper online for Figure 1b and ED Figure 5c,d.

Field-specific reporting

Please select the one below that is the best fit for your research. If you are not sure, read the appropriate sections before making your selection.

☒ Life sciences ☐ Behavioural & social sciences ☐ Ecological, evolutionary & environmental sciences

For a reference copy of the document with all sections, see nature.com/documents/nr-reporting-summary-flat.pdf

Life sciences study design

All studies must disclose on these points even when the disclosure is negative.

Sample size	Structure determination used all crystallographic and EM data available; other experiments were performed in triplicates which sufficed for statistical significance given the magnitude of the effects observed.
Data exclusions	chains D, E and F in the final crystallographic model showed poor electron density and where therefore excluded from structural analysis after structure determination, in compliance with established practice in the field.
Replication	We collected several crystallographic datasets which all showed the same data, to comparable resolutions. The kinetic traces shown in Fig.1 are three independent measurements on separate samples. Other experiments were performed in triplicates, all replications were successful
Randomization	A 5% 'test set' for cross-validation was defined by random selection of structure factors during the crystallographic analysis as per established practice. For EM, half-datasets were randomly defined for the purpose of Fourier shell correlation calculations, also as per established practice in the field. For other experiments samples were randomly allocated.
Blinding	The random selection of test-set reflections for crystallographic cross-validation as well as the allocation to half-datasets for Fourier shell correlation calculation were performed automatically, well before refinement, and structure refinement was done automatically as well. The investigators had no influence over either process, nor where they able to influence the results by 'cherry picking' the data before these procedures started. For other experiments no blinding was necessary as the magnitude of the effect observed precluded inadvertent human influence over the interpretation of the results.

Reporting for specific materials, systems and methods

We require information from authors about some types of materials, experimental systems and methods used in many studies. Here, indicate whether each material, system or method listed is relevant to your study. If you are not sure if a list item applies to your research, read the appropriate section before selecting a response.

Materials & experimental systems

n/a	Involved in the study	
☒	☐	Antibodies
☒	☐	Eukaryotic cell lines
☒	☐	Palaeontology and archaeology
☒	☐	Animals and other organisms
☒	☐	Human research participants
☒	☐	Clinical data
☒	☐	Dual use research of concern

Methods

n/a	Involved in the study	
☒	☐	ChIP-seq
☒	☐	Flow cytometry
☒	☐	MRI-based neuroimaging
