# Supplementary material for: Structural and functional characterization of the intracellular filament-forming nitrite oxidoreductase multiprotein complex
Source: Nat Microbiol. 2021 Jul 15;6(9):1129–39. doi: 10.1038/s41564-021-00934-8 (PMC8387239; doi:10.1038/s41564-021-00934-8)
Supplement: Supplementary file 4 — Full alignment of NxrA sequences. [file 41564_2021_934_MOESM4_ESM.pdf]

```
Kuenenia_stuttgartiensis
Kuenenia_stuttgartiensis      1  ...............MKLTRRAFLQVAGATGATLTL.AKNAMAFRLLKPAVVVDNPLDT
Jettenia_caeni                1  ...............MKLTRRTFLQVTGATGATFTL.ANKAMAFRLLKPAVEVGNPLDA
Brocadia_fulgida              1  ...............MKLTRRTFLQVAGATGATFTL.ADKAMAFRLLKPAVEVGNPLDA
Scalindua_brodae              1  ...............MKLTRRTFMKVAGGVTAAVSIPMRNALAFKSLKPAVEVDNPLDS
Nitrospira_defluvii           1  ..............MMQLSRRQFLKVSAGTVAVAAV.ADKALALTALQPVVEVNNPLGE
Nitrospira_nitrificans        1  ...............MLVSRRQFMKATAGTIAAAAV.ADKVLALTALQPVIEVGNPLGE
Nitrospira_inopinata          1  ...............MFLSRRQFLKVSVGTVAAVAV.ADKVLALTALQPVIEVGNPLGE
Nitrospina_gracilis           1  ...............MRLNRRKFLQVSAGVASAMALSSKR..VGAQLKPVVKVGNPLES
Nitrotoga_sp.                 1  ..........MMEIKNEIGRRSFLKLSATAGL..AVMANNAFAASPFLKPYVVDNPLKS
Hydrogenobaculum_sp.          1  ................MISRRDFLKNGS.VFL..AALSTPGFGKKLFEPLVIVGNPLAS
Beggiatoa_sp.                 1  .......................................MGW......IQDIIN
Thiocapsa_sp._KS1             1  ...........MSKTKD..D.....NGN.............GGSRW......FRE..L
Nitrolancea_hollandica_Lb        ...........................................................
Nitrobacter_winogradskyi      1  .......................................MSW......ILDLVN
Nitrobacter_hamburgensis      1  .......................................MSW......ILDLVN
Nitrococcus_mobilis           1  .......................................MGW......IQDLVN
Escherichia_coli              1  ...........MSKFLD..RFRYFKQKG...........ETFAD.....GHGQLL
Aromatoleum_aromaticum        1  MTRDEMISVEPEAAELQDQHRRDFLKRSGAAVLSLSLSSLATGVVPGFLK..DAQAGTKA

                                         α1            β1                  β2        β3        β4
Kuenenia_stuttgartiensis
Kuenenia_stuttgartiensis     44  YPDRRWESVYRDQYQYDRTFTYCCSPND..THACRIRAFVRNNVMMRVEQNYDHQNYSDL
Jettenia_caeni               44  YPDRAWESVYREQYRYDRTFTYCCSPND..THACRVRAFVRNEVLMRVEQNYDHQNYADL
Brocadia_fulgida             44  YPDRSWESVYRDQYRYDRTFTYTCSPND..THACRVRAFVRNEVLMRVEQNYDHQNYSDL
Scalindua_brodae             45  YPDRNWEDVYRNQYKYDRSFPFVCSPND..THQCRVRAFVRNGVVIRVEQDYEHQDYGDL
Nitrospira_defluvii          45  YPDRSWERVYHDQYRYDSSFTWVCSPND..THACRIRAFVRNGVVMRVEQNYDHQTYEDL
Nitrospira_nitrificans       44  YPDRSWERVYHDQYRYDSSFTWVCSPND..THACRIRAFVRNGVVMRVEQNYDHQTYEDL
Nitrospira_inopinata         44  YPDRSWERVYHDQYRYDSSFTWVCSPND..THACRVRAFVRNGVVMRVEQNYDHQTYEDL
Nitrospina_gracilis          43  YPDRRWEEVYRDQYKYERSFTYCCSPND..THQCRVRGFVRNGILMRIEQNYDHHKIRDL
Nitrotoga_sp.                48  YPNRDWEKVYRDMFHVDSEYIFLCAPND..THNCLLKAHVKNDVVIRISPSYGYGDAEDM
Hydrogenobaculum_sp.         41  YPNRGWEKIYRDIYKPDETTVILCNPND..THGCYLNAYVKNGIITRLEPTYKYGDATDI
Beggiatoa_sp.                10  PKTREWEQFYRTRWQYDKIVRSTHGVNC..TGGCSWRVYVKCGVITWEMQQVDYPKLEK.
Thiocapsa_sp._KS1            20  DEPRKWEDFYRRRWQYDKSVRTSHSVNC..SGSCSWEVFVKNGMITWELQKTDWPQIND.
Nitrolancea_hollandica_Lb     1  ...RQWEEFYRNRWQHDNITRSTHGVNC..TGGCSWMVFVKDGIITWEMQATDYPLLEP.
Nitrobacter_winogradskyi     10  PRERKWEEFYRNRWSHDNVFRSTHGVNC..TGGCSWAIYVKDGIITWEMQQTDYPLLER.
Nitrobacter_hamburgensis     10  PRERKWEDFYRNRWSHDNVFRSTHGVNC..TGGCSWAIYVKDGIITWEMQQTDYPLLER.
Nitrococcus_mobilis          10  PKTRRWEEFYRNRWQHDNIFRSTHGVNC..TGGCSWAIYVKDGIITWEMQQTDYPLLGRG
Escherichia_coli             27  NTNRDWEDGYRQRWQHDKIVRSTHGVNC..TGSCSWKIYVKNGLVTWETQQTDYPRTRP.
Aromatoleum_aromaticum       59  PGYASWEDIYRKEWKWDKVNWGSHLNICWPQGSCKFYVYVRNGIVWREEQAAQTPACNV.

                                  β5          α2              β6     α3
Kuenenia_stuttgartiensis
Kuenenia_stuttgartiensis    102  YGNKATRNWNPRMCLKGYTFHRRVYGPYRLRYPLIRKGWKR.WADDGF..........PE
Jettenia_caeni              102  YGNKATRNWNPRMCLKGYTFHRRVYGPYRLRYPLIRKGWKQ.WADDGF..........PE
Brocadia_fulgida            102  YGNKATRNWNPRMCLKGYTFHRRVYGPYRLRYPLIRRGWKQ.WADDGF..........PE
Scalindua_brodae            103  EGRKPQRSWNPRMCLKGFTFFRRVYGPHRLRFPLLRRGWKQ.WADDGF..........PE
Nitrospira_defluvii         103  YGNRGTFAHNPRMCLKGFTFHRRVYGPYRLKGPLMRKGWKQ.WMDDGS..........PE
Nitrospira_nitrificans      102  YGNRGTFAHNPRMCLKGFTFHRRVYGPYRLKGPLMRKGWKQ.WADDGS..........PE
Nitrospira_inopinata        102  YGNRGTFAHNPRMCLKGFTFHRRVYGPYRLKGPLMRKGWKQ.WMDDGA..........PE
Nitrospina_gracilis         101  YGNQADAAWNPRMCLRGMTFPRRVYGPYRNKYPMLRVGWKQ.WADDGF..........PY
Nitrotoga_sp.               106  DGNRSSHRWEPRICNKGMVMNRKAYSDRRPKGAMVRTGFKA.WADAGY..........PR
Hydrogenobaculum_sp.         99  YGIKASHRWEPRCCNKGLALVRRFYGDRRIRGPFVRKGYYE.WYKADF..........PR
Beggiatoa_sp.                67  ....SLPPYEPRGCQRGISASWYVYSPIRVRYPYVRGALVELWRRARQKYDDPVEAWESI
Thiocapsa_sp._KS1            77  ....ETPNYEPRGCQRGISASWYPYSPVRPKYPYVRAVLLDFWRAERAAGKDPIAAWGAI
Nitrolancea_hollandica_Lb    55  ....SLPPYEPRGCQRGISASWYVYSPIRVKYPYMRGPLMDFWREARAQHSDPIQAWASI
Nitrobacter_winogradskyi     67  ....SLPPYEPRGCQRGISASWYVYSPIRVKYPYIRGPLADLWHEAKASYPDPVQAWASL
Nitrobacter_hamburgensis     67  ....SLPPYEPRGCQRGISASWYVYSPIRIKYPYVRGPLLDMWREAKASSADPVQAWGAL
Nitrococcus_mobilis          68  EGGRGIPPYEPRGCQRGISASWYVYSPIRVKYPYARGVLLDFWREARSSHNNPVEAWSSI
Escherichia_coli             84  ....DLPNHEPRGCPRGASYSWYLYSANRLKYPMMRKRLMKMWREAKALHSDPVEAWASI
Aromatoleum_aromaticum      118  ....DYVDYNPLGCQKGSAFNNNLYGDERVKYPLKRV.......................

                                      α4                  α5                   α6
Kuenenia_stuttgartiensis
Kuenenia_stuttgartiensis    151  LTPENKTKYMFDNRGNDELLRASWDEAFTYASKGIIHITKKYSGPEGAQKLIDQ.GYPKE
Jettenia_caeni              151  LTPENKSKYMFDARGQDELLKASWDDAWTYAAKGIIHITKKYSGEEGARKLLDQ.GYPKE
Brocadia_fulgida            151  LTPENKSKYMFDARGQDELLKASWDEAWTYAAKGIIHITKKYSGEEGAKKLIEQ.GYPKE
Scalindua_brodae            152  LDWNAREKYKFTTRGTDEQLKMSWDDIIDYVAKGMIAIARAYSGEEGKKRLLERDKYAPE
Nitrospira_defluvii         152  LTSDVKRKYKFDSRFLDDMVRVSWDTAFTYVAKGLIVIGTRYSGEAGARRLREQ.GYAPE
Nitrospira_nitrificans      151  LTPEAKRKYKFDSRFLDDMLRVSWDTAFTYAAKAMIVIATRYSGEAGARRLREQ.GYAPE
Nitrospira_inopinata        151  LTPETKRKYKFDSRFLDDMLRVSWDTAFTYAAKAMIIIATRYSGEAGARRLREQ.GYAPE
Nitrospina_gracilis         150  LDKENREKYKFTSRGTDEFVRMTWDDTFTYLAKGHVAVGKAYSGQRGAQRLKNE.GYQPE
Nitrotoga_sp.               155  TGANGFPDQKYLQRGKEPFIKLPWTEAYALAAGALENIARTYSGDKGAALLTRQ.GYDPE
Hydrogenobaculum_sp.        148  DENGKPPSKYFINRGKDEWLKVSWEEVSDIIAKSLINIAKTYSGEKGKAYLKAQ.GYPEE
Beggiatoa_sp.               123  VSDPDSRKTYQQARGKGGFRRSDWHEVLEIITASNVYTIKKYGPDRIV.......GFSP.
Thiocapsa_sp._KS1           133  VENPERSKAYRNARGKAGWRRTTWDEATEIIAAAKIYTIKEHGADHLA.......SFSP.
Nitrolancea_hollandica_Lb   111  VQDEQKRQRWQRARGKGGYRRATWDECLELVASALLYTAKKYGPDRVT.......AFAP.
Nitrobacter_winogradskyi    123  VEDEEKRNRIQKARGKGGFRRAKWEELIELIAASCLYTARKHGPDRVM.......GFSP.
Nitrobacter_hamburgensis    123  VGDEQKRARMQKARGKGGYRRAKWEELVELIAAASLHTARKHGPDRIM.......GFSP.
Nitrococcus_mobilis         128  VTDENKRKRWQKARGKGGFRRTSWDELLELIASACLYTAQKYGPDRVM.......GFSP.
Escherichia_coli            140  IEDADKAKSFKQARGRGGFVRSSWQEVNELIEASNVYTIKNYGPDRVA.......GFSP.
Aromatoleum_aromaticum      151  ..........GKRGEGKWKRVSWDEAAGDIADSIIDSFEAQGSDGFI.......LDAP.
```

```
                                    α7       α8   β7                α9                                β8
Kuenenia_stuttgartiensis
Kuenenia_stuttgartiensis      210  MVDRMQGAGTRTFKGRGGMGLLGVIGKY.GMYRFNN.CLAIVDAHNRGVGPDQALGGRNW
Jettenia_caeni                210  MVDAMKGAGTRTFKGRGGMGLLGVIGKY.GMYRFNN.SLALVDSHNRGVGPDKALGGRNW
Brocadia_fulgida              210  MVDAMKGAGTRTFKGRGGMGLLGVIGKY.GMYRFNN.TLALVDSHNRGVGPDKALGGRNW
Scalindua_brodae              212  TLTHWNGAGTRCFKNRGGMGLLGVIGKYMGMYRFSN.MLALLDANVRGVGADKAMGGRRF
Nitrospira_defluvii           211  MIEMMKGAGVRTFKHRAGMPILGMMGKH.ANTRFNNCVLPLLDSWIRKVNPDQAQGGRYW
Nitrospira_nitrificans        210  MIEMMKGAGVRCFKHRAGMPILGFIGKH.SNTRFNNSVLPLLDTWIRKVGPDQAQGGRYW
Nitrospira_inopinata          210  MIEMMKGAGTRCFKHRAGMPVLGIIGKM.GNTRMNGGINALLDTWIRKVGPDQAQGGRYW
Nitrospina_gracilis           209  MIEAMGGSGPRTFKYRGGMGLLGVVGKY.GIYRLAN.MVGLLDAIIRGRGPGQVLGGRAW
Nitrotoga_sp.                 214  MIASMHGCGMKTMKFRSGMAALGVLRIY.SLKRFAQ.GLALLDAYVRNVGPDEASGAKVL
Hydrogenobaculum_sp.          207  MIDTMAGAGTRTLKFRGSMPWLAVLRYV.GQYRMSN.MMALLDSNIRKVEPDKALGGVGW
Beggiatoa_sp.                 175  ..................IPAMSQLSFA.AGSRFLG.................LMGGHLL
Thiocapsa_sp._KS1             185  ..................IPAMSMVSFL.SGHRLSN.................LLGGTML
Nitrolancea_hollandica_Lb     163  ..................IPAYSYVSFG.AGARFIQ.................LFGGFHL
Nitrobacter_winogradskyi      175  ..................IPAMSMLSFA.AGTRFLS.................LFGGGLM
Nitrobacter_hamburgensis      175  ..................IPAMSMLSFA.AGTRFLS.................LMGGSLL
Nitrococcus_mobilis           180  ..................IPAMSMLSFA.AGQRFLQ.................LFGGATI
Escherichia_coli              192  ..................IPAMSMVSYA.SGARYLS.................LIGGTCL
Aromatoleum_aromaticum        192  ..................HVHAGSIAWG.AGFRMTY.................LMDGVSP
```

```
                                    α10      α11              α12     β9             α13
Kuenenia_stuttgartiensis
Kuenenia_stuttgartiensis      268  SNYTWHGDQAPGHPFSHGLQTSDVDMNDVRFSKLLIQTGKNLIENKMPEAHWVTEVMERG
Jettenia_caeni                268  SNYTWHGDQAPGHPFSHGLQTSDVDMNDVRFSKLVIQTGKNLIENKMPEAHWLTQVFERG
Brocadia_fulgida              268  SNYTWHGDQAPGHPFVHGLQTSDVDMNDIRFSKLVIQTGKNLIENKMPEAHWLTEVMERG
Scalindua_brodae              271  SNYTWHGDQAPGHPFVHGLQASDVDFADLRYSKLVIQVGKNLIENKMPEAHWLTEVIERG
Nitrospira_defluvii           270  NNYTWHGDQDPSQPWWNGTQNCDVDLSDMRFTKLNTSWGKNFVENKMPEAHWKLESMERG
Nitrospira_nitrificans        269  NNYTWHGDQDPSQPFWNGTQNCDVDLSDMRFTKFNTSWGKNFVENKMPEAHWKLESIERG
Nitrospira_inopinata          269  SNYTWHGDQNPAHPWWSGVQGSDVDLSDMRFSKLNTSWGKNFVENKMPEAHWKLECIERG
Nitrospina_gracilis           267  SNYTWHGDQAPGHSWTHGMQTSDIDFADHRYAKLTIQWGKNLIENKMPEAHWYTEIMERG
Nitrotoga_sp.                 272  DSYSWHTDLAPGCPMVTGHQMLDYEFMVYEHAKLIVFWGNNFICTKMPDLHWVSESRLKG
Hydrogenobaculum_sp.          265  DNYSEHTDLPPAHTLVTGQQTVDFDLVTWEHAKLIVLWGMNPYGTKMPDSHWLTEAQIKG
Beggiatoa_sp.                 199  SFYDWYSDLPPAEPEVWGEQTDAHESADWYNAKFIVSMGSNLNMTRTPDVHFIAEARHNG
Thiocapsa_sp._KS1             209  SFYEWYHDLPHVMPMIWGDQTDVHESADWYQSAYWIVMGSNLPMTRTADAHFASEHKYNG
Nitrolancea_hollandica_Lb     187  SFYDWYADLPNSFPEVWGDQTDVCESADWYNSKFIVSNAANLNMTRTADVHFVSEARHNG
Nitrobacter_winogradskyi      199  SFYDWYADLPTSFPEIWGDQTDVCESADWYNSKFIVSMASNMNMTRTPDVHFISEARTEG
Nitrobacter_hamburgensis      199  SFYDWYADLPTSFPEIWGDQTDVCESADWYNSKFIVSMASNLNMTRTPDVHFIAEARTEG
Nitrococcus_mobilis           204  SFYDWYADLPNSFPEIWGDQTDVCESADWYNSKFIVSMASNLNMTRTPDVHFISEARHEG
Escherichia_coli              216  SFYDWYCDLPPASPQTWGEQTDVPESADWYNSSYIIAWGSNVPQTRTPDAHFFTEVRYKG
Aromatoleum_aromaticum        216  DINVDIGDTYMGAFHTFGKMHMGYSADNLLDAELIFMTCSNWSYTYPSSYHFLSEARYKG
                                          ★                                  ●
```

```
                                    β10         α14   β11             α15                α16
Kuenenia_stuttgartiensis
Kuenenia_stuttgartiensis      328  GKIVVITPEYSPSAQKADYWIPIRNN.TDTALFLGITKILIDNKWYDAD......YVKKF
Jettenia_caeni                328  GKLVVITPEYSPSAQKADYWIPIKCN.TDTALFLGITRILMDEKLYDAD......YVKKF
Brocadia_fulgida              328  GKLVVITPEYSPSAQKADYWIPVRCN.TDTALFLGLTKILMDEKLYDAD......YVKKF
Scalindua_brodae              331  GKLVVITPEYSPSATKADYWIPCRTGISDTTIFLTMTKIIMDNKWYDTD......FVKKF
Nitrospira_defluvii           330  ARLVIITPEYNPTASRADYWIPVRPE.TDGALFLGASKIILDENYQDID......FIKGF
Nitrospira_nitrificans        329  ARIAVITPEYNPTAQRADYWIPLRPQ.SDGALFLGACKIILDENMQDID......YLKQF
Nitrospira_inopinata          329  ARVVVITPEYNPTAYRADYWMPLRPQ.SDGALFLGAMKIIVDENMHDVD......FLKSF
Nitrospina_gracilis           327  GTLVTVCPEYNPPSTKADYWIPCRAGASDISIFLGCAKIIMDEGLVDVD......YVKDY
Nitrotoga_sp.                 332  CHIVDISIDYHAMSNKADDVIIIRPG.TDPALGLGCCHILIKNNWYDEN......YLKAN
Hydrogenobaculum_sp.          325  IKVIVISNDFMATARTADKVVITRTA.TDGALALSMAYVIMKEKLYDEN......FVKSF
Beggiatoa_sp.                 259  SKFVVFAPDFSQVAKYSDWWVPVHAG.QDGAFWVAVNHVILTEYYVKKETPSFNAYMKQF
Thiocapsa_sp._KS1             269  GKLVNLSPDYSDVTKFADLWVPVRPG.TDTAFILSCIHVILQEFHVNRRSEYFHSYVAQY
Nitrolancea_hollandica_Lb     247  TKFVVLAPDFSQVAKYSDWWIPVKAG.QDQALWMGADHVILKEFYIDRQVPYFIEYQKRY
Nitrobacter_winogradskyi      259  TKFVVLSPDFSQIAKYCDEWIPIQAG.QDTALWMAANHVILKEYYIDRQVPYFIDYVKRY
Nitrobacter_hamburgensis      259  TKFVVLSPDFSQIAKYCDEWIPIQAG.QDTALWMAANHVILKEYYVDRQVPYFIDYVKRY
Nitrococcus_mobilis           264  AKFVVLSPDFSQVSKYADWWIPVKKG.QDLGIWMAVGHVIYTEFYVKRQVPYFIDYVTRY
Escherichia_coli              276  TKTVAVTPDYAEIAKLCDLWLAPKQG.TDAAMALAMGHVMLREFHLDNPSQYFTDYVRRY
Aromatoleum_aromaticum        276  AEVVVIAPDFNPTTPAADLHVPVRVG.SDAAFWLGLSQVMIDEKLFDRQ......FVCEQ
```

```
                                      β12          β13                      α17          α18
Kuenenia_stuttgartiensis
Kuenenia_stuttgartiensis      381  TDFPLLIRTD........TLKRVSPKDIIPNYKLQDISDGPSYHIQGLK...........
Jettenia_caeni                381  TDFPLLVRTD........TLKRLQAKDIFPDYKLEDISHGASYKIHGLH...........
Brocadia_fulgida              381  TDFPLLVRTD........TLKRLQAKDVFLNYQLEDISQGASFKIHGLH...........
Scalindua_brodae              385  TDFPLLVRTD........TLKRLQPHEIIKDYKQPELDY..SHSVQGLT...........
Nitrospira_defluvii           383  TDMPLLVRTD........TLQYLDPHEVLKDYQVPDFTKSYSGRVQGLS...........
Nitrospira_nitrificans        382  TDMPLLVRTD........TLQYLDPRDVIQDYKFPDFSHSYSGRIQALK...........
Nitrospira_inopinata          382  TDAPILVRTD........TLQYLDPRDVIPDYKFPDFSKSYSGRIQSLK...........
Nitrospina_gracilis           381  TDMPLLVRTD........NLIRLHPDDYIPGYKNQPLPKD.GFTTKWMK...........
Nitrotoga_sp.                 385  TDMPLLIRTD........NWKNLKASDIIADYKLADLTHHLKVMKKGEKPTLPVAFQATA
Hydrogenobaculum_sp.          378  TDLPLLVRMD........NAKLLRASDIIPNYIPKALNQAVVYNPSKQMPPPPMKQE.KQ
Beggiatoa_sp.                 318  TDSPFLVVLN.QGEEGYEAGRLLRANTI.EAYKDEENGEW....................
Thiocapsa_sp._KS1             328  TNLPFLVILD.QEGDHFASGRFLRASDV.AAYADEELADW....................
Nitrolancea_hollandica_Lb     306  TDGPMLVKVHQTKGGAYSMGQFLRANRI.NRYKDVENGNW....................
Nitrobacter_winogradskyi      318  TDLPFLVELE.PNGNTYKTGRLLRSNRV.ARYKDVENGEW....................
Nitrobacter_hamburgensis      318  TDLPFLVELE.PNGTTYKTGRLLRARHV.PRYKDVENGDW....................
Nitrococcus_mobilis           323  TDMPFLVKLE.KDGDSYRPGRYLTSEEV.KKYKKQENAAW....................
Escherichia_coli              335  TDMPMLVMLE.ERDGYYAAGRMLRAADLVDALGQENNPEW....................
Aromatoleum_aromaticum        329  TDLPLLVRMD........TGKFLSAEDVDG.....GEAK.....................
```

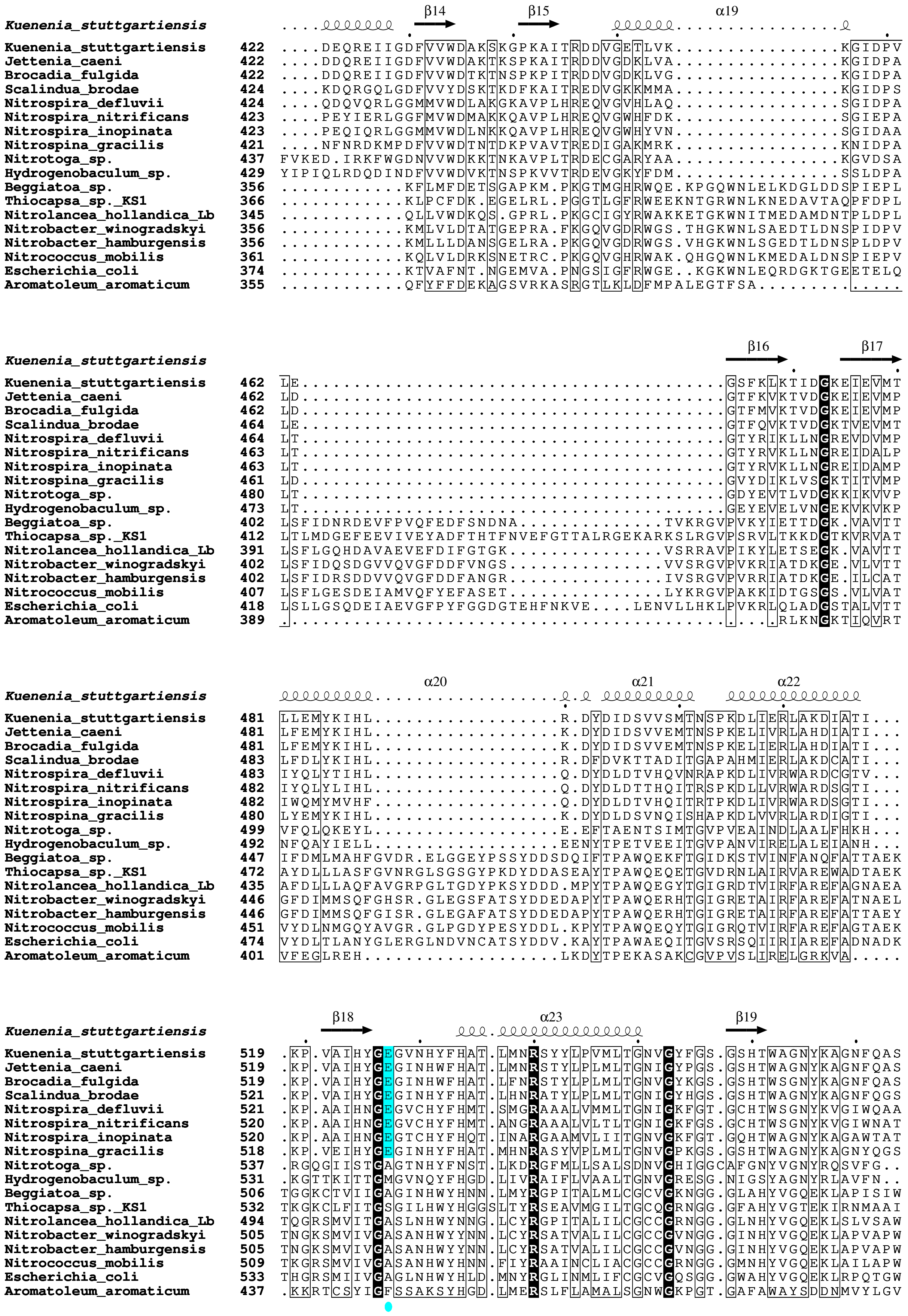
β14 β15 α19
Kuenenia_stuttgartiensis
Kuenenia_stuttgartiensis 422 ....DEQREIIGDFVVWDAKSKGPKAITRDDVGETLVK...............KGIDPV
Jettenia_caeni 422 ....DDQREIIGDFVVWDAKTKSPKAITRDDVGDKLVA...............KGIDPA
Brocadia_fulgida 422 ....DDQREIIGDFVVWDTKTNSPKPITRDDVGDKLVG...............KGIDPA
Scalindua_brodae 424 ....KDQRGQLGDFVVYDSKTKDFKAITREDVGKKMMA...............KGIDPS
Nitrospira_defluvii 424 ....QDQVQRLGGMMVWDLAKGKAVPLHREQVGVHLAQ...............SGIDPA
Nitrospira_nitrificans 423 ....PEYIERLGGFMVWDMAKKQAVPLHREQVGWHFDK...............SGIEPA
Nitrospira_inopinata 423 ....PEQIQRLGGMMVWDLNKKQAVPLHREQVGWHYVN...............SGIDAA
Nitrospina_gracilis 421 ....NFNRDKMPDFVVWDTNTDKPVAVTREDIGAKMRK...............KNIDPA
Nitrotoga_sp. 437 FVKED.IRKFWGDNVVWDKKTNKAVPLTRDECGARYAA...............KGVDSA
Hydrogenobaculum_sp. 429 YIPIQLRDQDINDFVVWDVKTNSPKVVTRDEVGKYFDM...............SSLDPA
Beggiatoa_sp. 356 ...........KFLMFDETSGAPKM.PKGTMGHRWQE.KPGQWNLELKDGLDDSPIEPL
Thiocapsa_sp._KS1 366 ...........KLPCFDK.EGELRL.PGGTLGFRWEEKNTGRWNLKNEDAVTAQPFDPL
Nitrolancea_hollandica_Lb 345 ...........QLLVWDKQS.GPRL.PKGCIGYRWAKKETGKWNITMEDAMDNTPLDPL
Nitrobacter_winogradskyi 356 ...........KMLVLDTATGEPRA.FKGQVGDRWGS.THGKWNLSAEDTLDNSPIDPV
Nitrobacter_hamburgensis 356 ...........KMLLLDANSGELRA.PKGQVGDRWGS.VHGKWNLSGEDTLDNSPLDPV
Nitrococcus_mobilis 361 ...........KQLVLDRKSNETRC.PKGQVGHRWAK.QHGQWNLKMEDALDNSPIEPV
Escherichia_coli 374 ...........KTVAFNT.NGEMVA.PNGSIGFRWGE..KGKWNLEQRDGKTGEETELQ
Aromatoleum_aromaticum 355 ...........QFYFFDEKAGSVRKASRGTLKLDFMPALEGTFSA..............
β16 β17
Kuenenia_stuttgartiensis
Kuenenia_stuttgartiensis 462 LE..................................GSFKLKTIDGKETEVMT
Jettenia_caeni 462 LD..................................GTFKVKTVDGKEIEVMP
Brocadia_fulgida 462 LD..................................GTFMVKTVDGKEIEVMP
Scalindua_brodae 464 LE..................................GTFQVKTVDGKTVEVMT
Nitrospira_defluvii 464 LT..................................GTYRIKLLNGREVDVMP
Nitrospira_nitrificans 463 LT..................................GTYRVKLLNGREIDALP
Nitrospira_inopinata 463 LT..................................GTYRVKLLNGREIDAMP
Nitrospina_gracilis 461 LD..................................GVYDIKLVSGKTITVMP
Nitrotoga_sp. 480 LT..................................GDYEVTLVDGKKIKVVP
Hydrogenobaculum_sp. 473 LT..................................GEYEVELVNGEKVKVKP
Beggiatoa_sp. 402 LSFIDNRDEVFPVQFEDFSNDNA.............TVKRGVPVKYIETTDGK.VAVTT
Thiocapsa_sp._KS1 412 LTLMDGEFEEVIVEYADFTHTFNVEFGTTALRGEKARKSLRGVPSRVLTKKDGTKVRVAT
Nitrolancea_hollandica_Lb 391 LSFLGQHDAVAEVEFDIFGTGK..............VSRRAVPIKYLETSEGK.VAVTT
Nitrobacter_winogradskyi 402 LSFIDQSDGVVQVGFDDFVNGS..............VVSRGVPVKRIATDKGE.VLVTT
Nitrobacter_hamburgensis 402 LSFIDRSDDVVQVGFDDFANGR..............IVSRGVPVRRIATDKGE.ILCAT
Nitrococcus_mobilis 407 LSFLGESDEIAMVQFYEFASET..............LYKRGVPAKKIDTGSGS.VLVAT
Escherichia_coli 418 LSLLGSQDEIAEVGFPYFGGDGTEHFNKVE....LENVLLHKLPVKRLQLADGSTALVTT
Aromatoleum_aromaticum 389 ....RLKNGKTIQVRT
α20 α21 α22
Kuenenia_stuttgartiensis
Kuenenia_stuttgartiensis 481 LLEMYKIHL.................R.DYDIDSVVSMTNSPKDLIERLAKDIATI...
Jettenia_caeni 481 LFEMYKIHL.................K.DYDIDSVVEMTNSPKELIVRLAHDIATI...
Brocadia_fulgida 481 LFEMYKIHL.................K.DYDIDSVVEMTNSPKELIERLAHDIATI...
Scalindua_brodae 483 LFDLYKIHL.................R.DFDVKTTADITGAPAHMIERLAKDCATI...
Nitrospira_defluvii 483 IYQLYTIHL.................Q.DYDLDTVHQVNRAPKDLIVRWARDCGTV...
Nitrospira_nitrificans 482 IYQLYLIHL.................Q.DYDLDTTHQITRSPKDLLVRWARDSGTI...
Nitrospira_inopinata 482 IWQMYMVHF.................Q.DYDLDTVHQITRTPKDLIVRWARDSGTI...
Nitrospina_gracilis 480 LYEMYKIHL.................K.DYDLDSVNQISHAPKDLVVRLARDIGTI...
Nitrotoga_sp. 499 VFQLQKEYL.................E.EFTAENTSIMTGVPVEAINDLAALFHKH...
Hydrogenobaculum_sp. 492 NFQAYIELL.................EENYTPETVEEITGVPANVIRELALEIANH...
Beggiatoa_sp. 447 IFDMLMAHFGVDR.ELGGEYPSSYDDSDQIFTPAWQEKFTGIDKSTVINFANQFATTAEK
Thiocapsa_sp._KS1 472 AYDLLLASFGVNRGLSGSGYPKDYDDASEAYTPAWQEQETGVDRNLAIRVAREWADTAEK
Nitrolancea_hollandica_Lb 435 AFDLLLAQFAVGRPGLTGDYPKSYDDD.MPYTPAWQEGYTGIGRDTVIRFAREFAGNAEA
Nitrobacter_winogradskyi 446 GFDIMMSQFGHSR.GLEGSFATSYDDEDAPYTPAWQERHTGIGRETAIRFAREFATNAEL
Nitrobacter_hamburgensis 446 GFDIMMSQFGISR.GLEGAFATSYDDEDAPYTPAWQERHTGIGRETAIRFAREFATTAEY
Nitrococcus_mobilis 451 VYDLNMGQYAVGR.GLPGDYPESYDDL.KPYTPAWQEQYTGIGRQTVIRFAREFAGTAEK
Escherichia_coli 474 VYDLTLANYGLERGLNDVNCATSYDDV.KAYTPAWAEQITGVSRSQIIRIAREFADNADK
Aromatoleum_aromaticum 401 VFEGLREH.................LKDYTPEKASAKCGVPVSLIRELGRKVA.....
β18 α23 β19
Kuenenia_stuttgartiensis
Kuenenia_stuttgartiensis 519 .KP.VAIHYGEGVNHYFHAT.LMNRSYYLPVMLTGNVGYFGS.GSHTWAGNYKAGNFQAS
Jettenia_caeni 519 .KP.VAIHYGEGINHWFHAT.LMNRSTYLPLMLTGNIGYPGS.GSHTWAGNYKAGNFQAS
Brocadia_fulgida 519 .KP.VAIHYGEGINHWFHAT.LFNRSTYLPLMLTGNVGYKGS.GSHTWSGNYKAGNFQAS
Scalindua_brodae 521 .KP.VAIHYGEGINHYFHAT.LHNRATYLPLMLTGNIGYHGS.GSHTWAGNYKAGNFQGS
Nitrospira_defluvii 521 .KP.AAIHNGEGVCHYFHMT.SMGRAAALVMMLTGNIGKFGT.GCHTWSGNYKVGIWQAA
Nitrospira_nitrificans 520 .KP.AAIHNGEGVCHYFHMT.ANGRAAALVLTLTGNIGKFGS.GCHTWSGNYKVGIWNAT
Nitrospira_inopinata 520 .KP.AAIHNGEGTCHYFHQT.INARGAAMVLIITGNVGKFGT.GQHTWAGNYKAGAWTAT
Nitrospina_gracilis 518 .KP.VEIHYGEGINHYFHAT.MHNRASYVPLMLTGNVGPKGS.GSHTWAGNYKAGNYQGS
Nitrotoga_sp. 537 .RGQGIISTGAGTNHYFNST.LKDRGFMLLSALSDNVGHIGGCAFGNYVGNYRQSVFG..
Hydrogenobaculum_sp. 531 .KGTTKITTGMGVNQYFHGD.LIVRAIFLVAALTGNVGRESG.NIGSYAGNYRLAVFN..
Beggiatoa_sp. 506 TGGKCTVIIGAGINHWYHNN.LMYRGPITALMLCGCVGKNGG.GLAHYVGQEKLAPISIW
Thiocapsa_sp._KS1 532 TKGKCLFITGSGILHWYHGGSLTYRSEAVMGILTGCQGRNGG.GFAHYVGTEKIRNMAAI
Nitrolancea_hollandica_Lb 494 TQGRSMVITGASLNHWYNNG.LCYRGPITALILCGCCGRNGG.GLNHYVGQEKLSLVSAW
Nitrobacter_winogradskyi 505 TNGKSMVIVGASANHWYYNN.LCYRSATVALILCGCCGVNGG.GINHYVGQEKLAPVAPW
Nitrobacter_hamburgensis 505 TNGKSMVIVGASANHWYYNN.LCYRSATVALILCGCCGVNGG.GINHYVGQEKLAPVAPW
Nitrococcus_mobilis 509 TKGRSMVIVGASANHWYHNN.FIYRAAINCLIACGCCGRNGG.GMNHYVGQEKLAILAPW
Escherichia_coli 533 THGRSMIIVGAGLNHWYHLD.MNYRGLINMLIFCGCVGQSGG.GWAHYVGQEKLRPQTGW
Aromatoleum_aromaticum 437 .KKRTCSYIGFSSAKSYHGD.LMERSLFLAMALSGNWGKPGT.GAFAWAYSDDNMVYLGV

Kuenenia_stuttgartiensis

```
Kuenenia_stuttgartiensis       575 ......................................................KWSGPG
Jettenia_caeni                 575 ......................................................KWSGPG
Brocadia_fulgida               575 ......................................................KWSGPG
Scalindua_brodae               577 ......................................................KWSGPG
Nitrospira_defluvii            577 ......................................................PWSGAG
Nitrospira_nitrificans         576 ......................................................PWSGVG
Nitrospira_inopinata           576 ......................................................PWSGAG
Nitrospina_gracilis            574 ......................................................HWSGPG
Nitrotoga_sp.                  593 ...........................................................G
Hydrogenobaculum_sp.           586 ...........................................................G
Beggiatoa_sp.                  564 ...................................KTVAMAADWGSAARLQNAPTWHYIH
Thiocapsa_sp._KS1              591 ...................................GTIGNAADWGNVGRQQNSTSYFYFH
Nitrolancea_hollandica_Lb      552 ...................................QSWALAKDWFPPSRVQQSSTWHYAH
Nitrobacter_winogradskyi       563 ...................................STVAMALDWNKPPRVVQSSTWHYAH
Nitrobacter_hamburgensis       563 ...................................ASIALALDWSKPPRVVQSSTWHYAH
Nitrococcus_mobilis            567 ...................................STLTSALDWGVKPRLTQSATWHYSN
Escherichia_coli               591 ...................................QPLAFALDWQRPARHMNSTSYFYNH
Aromatoleum_aromaticum         494 MSKPTAQGGMDELHQMAEGFNKRTLEADPTSTDEMGNIEFMKVVTSAVGLVPPAMWLYYH
```

Kuenenia_stuttgartiensis

```
Kuenenia_stuttgartiensis       581 FYGWVAEDVFKPNLDPYA.....................................SAKDL
Jettenia_caeni                 581 FYGWVAEDVFNPNLDPDA.....................................PAMDL
Brocadia_fulgida               581 FYGWVAEDVFNPNLDPNA.....................................PAMDL
Scalindua_brodae               583 FKGIIAEDPFDMNLDPHA.....................................DGKNI
Nitrospira_defluvii            583 ASVYLGEDPWNLNLRDDV.....................................HGKEI
Nitrospira_nitrificans         582 GGVHLSEDPWHINLDANA.....................................HGKEI
Nitrospira_inopinata           582 LSVHTGEDPFNITLDPNA.....................................HGKEI
Nitrospina_gracilis            580 FASMVAEDPFNPVLDISK.....................................NVDWA
Nitrotoga_sp.                  594 FGQYLAEDPFNPELDGRK.....................................MV..T
Hydrogenobaculum_sp.           587 VGQWNAEDPFNIELDPSK.....................................L...A
Beggiatoa_sp.                  589 CDQWRYEGPFAKYSSVKGDNE.WTGG.HAADNNARAVRMGWLPFYPQFE.RSSLEIVKDA
Thiocapsa_sp._KS1              616 TDQWRYDGMSLDPLWAPRAKEMPARANHAADMNAVAVRNGWLPFFPQFDERNPTDVMQDA
Nitrolancea_hollandica_Lb      577 SDQWRYEGDFTDYAPIPRETR.WAKG.HAMDLLASAVRMGWMPMYPQFN.RNPLELVKQA
Nitrobacter_winogradskyi       588 SCQWRYEQEFTEYGLTAPNPR.WAKG.HAIDLEAKSVRCGWMPFTPNFH.RNPIEVVAEA
Nitrobacter_hamburgensis       588 SCQWRYEQEFTEYGLTAPNPR.WAKG.HAIDLEAKAVRSGWMPFTPHFN.RNPIEVAAEA
Nitrococcus_mobilis            592 SDSWRYDGLAEEYANLPPHSK.LAKGQHSIDLIAKSVRMGWMPYYPQFD.RSPHEVARAA
Escherichia_coli               616 SSQWRYETVTAEELLSPMADK.SRYTGHLIDFNVRAERMGWLPSAPQLG.TNPLTIAGEA
Aromatoleum_aromaticum         554 VG..YDQLWNNKAWTDPALK........................................
```

Kuenenia_stuttgartiensis

```
                                          α24         β20     β21              β22
Kuenenia_stuttgartiensis       604 NIKGRALDEEVAYWNHSERPLIVNTPKYGRKVFTGKTHMPSP...TKVLWFTNVN.LINN
Jettenia_caeni                 604 KVKGRAYDEEVAYWNHNDRPLIVDTPKYGRKCFTGKTHMPTP...TKIMWFTNVN.LVNN
Brocadia_fulgida               604 KVKGRAYDEEVAYWNHNDRPLIVNTPKYGRKCFTGKTHMPTP...TKIMWFTNVN.LVNN
Scalindua_brodae               606 HAHSYAMDEEVAYWNHGDTPLIVNTPKFGRKSFTGETHYNTP...TKVLWFNNVN.LINN
Nitrospira_defluvii            606 KYRKYYYGEEPGYWNHGDNALIVNTPKYGRKVFTGKTHMPSP...SKVRWVVNVN.ILNN
Nitrospira_nitrificans         605 KYRNYYYGEEPAYWNHGDTALIVNTPKYGRKVFTGKTHMPTP...SKFRWVVNVN.LLNN
Nitrospira_inopinata           605 HTRSYYYGEEVGYWNHGDTALIVNTPKYGRKVFTGKTHMPTP...SKFRWVTNVN.VVNN
Nitrospina_gracilis            603 NVKGYLKGEEVGYWAHRDKALIVNTPRYGRKVFTGRTHMPTP...SKLIWFVNVN.VINN
Nitrotoga_sp.                  615 KLAHYTDDESAHYYNYGDRPLRN.....GTRLLTDPGHMPAP...TKVLWQANSNSSLGN
Hydrogenobaculum_sp.           607 KVKFYWKGESQHYFADGDRPLKV.....GDKMLTGKTHMPVP...TKFMWMADNNSGLGN
Beggiatoa_sp.                  646 EQAGAQTDEEVVQYVVDQ.......LK.ERKLRFAAEDPDHPNNWPRVWFIWRGNAILAS
Thiocapsa_sp._KS1              676 RKAGCKSLEDVKTWVAQQ.......FK.ERKLKFALPNVDAPKNHPKVLWIYRGNLIGTS
Nitrolancea_hollandica_Lb      634 EAAGAKSDEQVVEWVVGQ.......LK.SGNLRYAIEDVDAPENWPRVWLIWRGNAFQSS
Nitrobacter_winogradskyi       645 ERAGAKSTADIATYVADQ.......VA.SKKLDLAINDPDAEENWPRVWFIWRANAIQSS
Nitrobacter_hamburgensis       645 ERAGAKSTEDIATHVIDQ.......VA.SKKLNLAIEDPDAAENWPRLWFIWRGNAIQSS
Nitrococcus_mobilis            650 QAAGAKDDKGIVNYTVQA.......LK.KKELSFATDDPDAPENWPRVWFVWRGNAMQSS
Escherichia_coli               674 EKAGMN....PVDYTVKS.......LK.EGSIRFAAEQPENGKNHPRNLFIWRSNLLGSS
Aromatoleum_aromaticum         572 KSFGA.........YLDE.......AK.EKGWWTNDHIRPAPDKTPQVYMLLSQNPMRRK
```

Kuenenia_stuttgartiensis

```
                                                        α25                   β23
Kuenenia_stuttgartiensis       660 AKHVYQMLK..............................NVNPNIEQIMSTDIEITGSIE
Jettenia_caeni                 660 AKHVYQMLK..............................NVNPNIEQIMSNDIEMTGSIE
Brocadia_fulgida               660 AKHVYQMLK..............................NVNPNIEQIMSTDIEVTGSIE
Scalindua_brodae               662 AKWVYEMMK..............................NVNPRIDLIISSDIMMTSSIQ
Nitrospira_defluvii            662 AKHHYDMVK..............................NVDPNIEMLVTQDIEMTSDVN
Nitrospira_nitrificans         661 AKHHYDMVR..............................NVDPNIECLITQDIEMTSDVN
Nitrospira_inopinata           661 AKHHYDMVK..............................NVDPNIECLITQDIEMTSDIN
Nitrospina_gracilis            659 AKWFYELVF..............................NTNNNVDMIVAQDIEFTGSCE
Nitrotoga_sp.                  667 AKGHYDMVV..............................NTLPRWEAIFYSDWNWTASCE
Hydrogenobaculum_sp.           659 QKWAYNVMF..............................NVLPNVECVVTNDWWWSLSCE
Beggiatoa_sp.                  698 GKGHEYFLRHYLGTHSNAVADEEVSK..EMLQEVVWRDEAPVGKMDLVVDINFRMDTSAL
Thiocapsa_sp._KS1              728 MRGHELNLKHMLGTHNNVLGGETRAK..DMVNEIDWTENAPLGKLDLIYNVNLRMDSSAN
Nitrolancea_hollandica_Lb      686 AKGAEYFLRHYSGTHDNAVAEEH.AK..GRVKDVVFREPAPRGKFDLLCDLNFRMDTTAL
Nitrobacter_winogradskyi       697 AKGHEFFLRHYLGAHDNVIAEER.AK..GKTTTVKYRDTAPQGKYDLVVDINFRMNTTGL
Nitrobacter_hamburgensis       697 AKGHEFFLRHYLGTHDNAIAEDR.AK..GKTHTVKYHDTAPRGKYDLVVDLNFRMDTSSL
Nitrococcus_mobilis            702 AKGAEFFLRHYLGTHDNAVAEER.AK..PHVKHVKYREPAPRGKFDLVVDINFRMDSTAL
Escherichia_coli               722 GKGHEFMLKYLLGTEHGIQGKDLGQQGGVKPEEVDWQDNGLEGKLDLVVTLDFRLSSTCL
Aromatoleum_aromaticum         615 RSGAKM........................F......PDVLFPKLKMIFALETRMSSSAM
```

```
                                   β24          β25        β26                α26
Kuenenia_stuttgartiensis

Kuenenia_stuttgartiensis      690  YADFAFPANSWVEFQEFEITNSC.SNPFIQIWGKTGITPVYESKDDVKILAGMASKLGEL
Jetteia_caeni                 690  YADFAFPANSWAEFETHEITTSC.SNPFIQIWK.GGIKPVNDSKDDVMILAGMAAKLGEL
Brocadia_fulgida              690  YADFAFPANSWVEFETHEITSSC.SNPFVQIWK.GGIRPVNDTKDDVMILAGMATKLGEL
Scalindua_brodae              692  YADVGFPVNSWMEFEHYEVTCSC.SNPFLQVWK.GGIKPLHDTKDDVMVPALVSKRMGEL
Nitrospira_defluvii           692  HADVAFAVNSWMEFTYPEMTATV.SNPWVQIWK.GGIRPLYDTRNDLDSFAGVAAKLKEM
Nitrospira_nitrificans        691  HADIAFAVNSWMEFTYPEMTATV.SNPWVQIWK.GGIRPLYDTRNDADTFAGVAAKLAEI
Nitrospira_inopinata          691  HADIAFACNSWMEFTYPEMTITV.SNPWVQIWK.GGIRPLYDTRNDLDTFAGVAAKLSDM
Nitrospina_gracilis           689  YADFVLAANSWAEFESYEITSAC.SNPFHQIWGGTGIKPVFDTIDDNLIHREFAKRLSQV
Nitrotoga_sp.                 697  YSDIVWGVDSWLENKHTDMACSC.SNPFLTVSPITPLRRFQDTVGDAEVPAGIFRAFTAM
Hydrogenobaculum_sp.          689  YSDIILGVDAWDENRFWDISGSC.TNPFFLVWPRTKQKRLFDTKNDIETYALVAKRLSEL
Beggiatoa_sp.                 756  YSDIVLPTAHWYE..KNDINTTD.MHSFIHPLS.EAVAPNWEARTDWNIYKSIAKGVSQL
Thiocapsa_sp._KS1             786  YSDIVLPTAHWYE..KFDLTCTD.LHSFLHPFT.PAHDPAYESKHDWEAFRLIAEKISEL
Nitrolancea_hollandica_Lb     743  YSDIVLPTAMWYE..KNDLNTTD.LHTFIHPLG.AAVPPVWEAKSDYEIFKSLAKKVSEL
Nitrobacter_winogradskyi      754  YSDIILPTAFWYE..KNDLNTTD.LHSFLHVLG.QAVPPVWESKTDWEIFKLIAKKVSEL
Nitrobacter_hamburgensis      754  YSDIVLPTAFWYE..KNDLNTTD.LHSFLHVLG.QAVPPVWESKTDWDIFKLIAKKVSEL
Nitrococcus_mobilis           759  YSDIILPTAFWYE..KNDLNSTD.LHSFIHPLS.QAVPPVWESKSDWDIFKAFAKKISEL
Escherichia_coli              782  YSDIILPTATWYE..KDDMNTSD.MHPFIHPLS.AAVDPAWEAKSDWEIYKAIAKKFSEV
Aromatoleum_aromaticum        645  YADIVLPCAWYYE..KHEMTTPCSGNPFFTFVD.RSVAPPGECREEWDAIALILKKVGER

                                      α27
Kuenenia_stuttgartiensis                                          .............................

Kuenenia_stuttgartiensis      749  LRDKRFEDNWKFAI........EGRASVYIN.............................
Jettenia_caeni                748  LRDMRFHDVWKFAL........EGRPEVYIQ.............................
Brocadia_fulgida              748  LRDMRFRDYWKFAL........EGRPEVYIQ.............................
Scalindua_brodae              750  LGDRRFADYWKFAL........EDRPEVYMQ.............................
Nitrospira_defluvii           750  TGEQRMADTYKFVY........HNRVDIYVQ.............................
Nitrospira_nitrificans        749  TGEKRMRDVFHFVY........ENRVDVYAQ.............................
Nitrospira_inopinata          749  TGDKRMKDYFAMVY........ANRVDVYAQ.............................
Nitrospina_gracilis           748  TGDKRFADYVKVYEG....EAPNRTKAMIR..............................
Nitrotoga_sp.                 756  TGDQRFADFFKFVGTMPGGNPADRDSEVYGQ.............................
Hydrogenobaculum_sp.          748  TGDKRFKEYWHFVF........EEKPEVYIQ.............................
Beggiatoa_sp.                 812  AEKHLPEPVKDFVCIPL....EHDYPDELAQ.....PSIKDWAKGECEPIPGKTMPKMKV
Thiocapsa_sp._KS1             842  AKVHLPDPIEDLVMTAL....STDTPDEMAQPM...GELRDWWKGESEPIPGKTFPNVSV
Nitrolancea_hollandica_Lb     799  APSVFPQPVKDIVSNPL....AHDTPDELSQDYLAEMKVLDWGKGEIEMIPGKTAPHFKI
Nitrobacter_winogradskyi      810  SPLAFSKPVRDIVLQPL....MHDTPDELAQ.....PEILDWAEGECKLVPGKSFPHVRV
Nitrobacter_hamburgensis      810  APLAFSKPVRDVVLQPL....MHDTPDELAQ.....PEILDWAEGECKPVPGKSFPHVRV
Nitrococcus_mobilis           815  APSVFPGPVKDVFAGPL....MHDTPDQMAQ.....REVRDWMEGECEPIPGKTMPHFEV
Escherichia_coli              838  CVGHLGK.ETDIVTLPI....QHDSAAELAQP....LDVKDWKKGECDLIPGKTAPHIMV
Aromatoleum_aromaticum        702  AAARGLTE..............................................FNDHNG

                                                       α28
Kuenenia_stuttgartiensis           ............................................................

Kuenenia_stuttgartiensis           ............................................................
Jettenia_caeni                     ............................................................
Brocadia_fulgida                   ............................................................
Scalindua_brodae                   ............................................................
Nitrospira_defluvii                ............................................................
Nitrospira_nitrificans             ............................................................
Nitrospira_inopinata               ............................................................
Nitrospina_gracilis                ............................................................
Nitrotoga_sp.                      ............................................................
Hydrogenobaculum_sp.               ............................................................
Beggiatoa_sp.                 863  VVRDYKNLYNRFTSFGPDAREK.GIGAHGVHWHIDDMYDETLKT..........HPTTQW
Thiocapsa_sp._KS1             895  VTRDYTKIHDMYTTLGPAVTKPNSYGAKGIKGDLAEVYEQMKESY............LVG
Nitrolancea_hollandica_Lb     855  VERDYVNFYNRWISYGRKAREN.GIVGNGVHIDIKPFYDELLENPLDAQPDVRHMRCIEW
Nitrobacter_winogradskyi      861  VERDYANLYNKFISFGPKARED.GISAVGVNIPIKKQYDQMLENPIMPMPDSRHMRCVEW
Nitrobacter_hamburgensis      861  VERDYANLYNKFISFGPKARED.GVSAVGVNVPIKKQYDQMLDNPIMPMPDPRHMRCVEW
Nitrococcus_mobilis           866  FERDYSLIYNKYISLGPAVREK.GISGNGCNFPVTKQYDELCDQPVGGSPDPRHRRAIEW
Escherichia_coli              889  VERDYPATYERFTSIGPLMEKI.GNGGKGIAWNTQSEMDLLRKLNYT.........KAEG
Aromatoleum_aromaticum        716  RKRRYDELYKKFTMDGHLLTNEDCL............KEMVDI.................

                                                                        α29       β27
Kuenenia_stuttgartiensis           ............................................................

Kuenenia_stuttgartiensis      772  ...............................RLLDGSTTMKGYTCEDILN.GKYGEPGVA
Jettenia_caeni                771  ...............................RLLDGSTTTKGYSFVDIIN.GKYGEPGVA
Brocadia_fulgida              771  ...............................RLLDGSTTFKGYTFDDIVN.GKYGEPGVA
Scalindua_brodae              773  ...............................RLFDGSVPTRGYNVNDMIA.GKYGVPGGA
Nitrospira_defluvii           773  ...............................RILDASTTFFGYSADVMLK.SEK....GW
Nitrospira_nitrificans        772  ...............................RLLDASSTFYGYSADVLLK.SEK....GW
Nitrospira_inopinata          772  ...............................RMLDASSTFYGYSADVMLK.SEK....GW
Nitrospina_gracilis           774  ...............................RLFTTSTAGMGYNIDDIIN.GKYGEPGAC
Nitrotoga_sp.                 787  ...............................RVLNAGSATRGMVFAEIREKAKRGIP..T
Hydrogenobaculum_sp.          771  ...............................RIINASSNLKGYKIEEVAAKAEQGIP..S
Beggiatoa_sp.                 912  NGDTYPSLNEVVHAINLILRFAPETNGESAYRAFTAHEKKVGLPLKDLGE.GNRGITMTY
Thiocapsa_sp._KS1             943  EKQGRPNMETAAKVCEVILRISPESDGELSYQIFKNLEKRCGVPLAHIVE.PERETAYHY
Nitrolancea_hollandica_Lb     914  GGQRYPRLEFAEEACNLIMFLAPESNGEVCYHAFKEEEKQLGLPLADLAE.AVRGVRMNF
Nitrobacter_winogradskyi      920  GGKRYPSLEDVLDACNVVLLCAPVANGEVSYQGFVNEEQHVGLPLADIAE.PTRGVSSTF
Nitrobacter_hamburgensis      920  GGKRYPSLEDVLDGCNTVLLCAPEANGEVCYQAFHNEEHHVGLPLVDLAE.PTRNVATTF
Nitrococcus_mobilis           925  GGKRYPCLEDALDAANVLLYMAPETNGEVCYQAFKAEEHHTGVPLVDLAE.PYRGVRMTF
Escherichia_coli              939  PAKGQPMLNTAIDAAEMILTLAPETNGQVAVKAWAALSEFTGRDHTHLAL.NKEDEKIRF
Aromatoleum_aromaticum        747  ..............NRAVGVF....AKDYTYEKFKKEGQTRFLSM...GT.GV..SR..Y
```

```
                                                          α30                                          α31
Kuenenia_stuttgartiensis
Kuenenia_stuttgartiensis      800  M...LLFRTYPRHPFW..............EQVHE..SLPFYTPTGRLQAYNDEPEIIEY
Jettenia_caeni                799  L...LLYRTYPRHPFW..............EQVHE..SIPFYTPTGRLQGYNDESEIIEY
Brocadia_fulgida              799  L...LLYRTYPRQPFW..............EQVHE..SLPFYTPTGRMQAYNDESEIIEY
Scalindua_brodae              801  L...LNFGTYPRTPMY..............EQVVY..DRPFHTDDGRLHSYCDIPEAIEY
Nitrospira_defluvii           797  M...VMCRTYPRHPLW..............EETNE..SKPHWTRSGRLETYRIEPEAIEY
Nitrospira_nitrificans        796  M...VMVRTYPRHPLW..............EETNE..SKPMWTRSGRIESYRIEPEAIEY
Nitrospira_inopinata          796  M...VMVRTYPRHPFW..............EETNE..SKPMWTRSGRYENYRPEAEAIEY
Nitrospina_gracilis           802  L...LLYRTYPRSPFW..............EMYTE..SKPFYTPNGRIQFYNDEPEAIEW
Nitrotoga_sp.                 814  I...FMARTYPRVSGW..............EQTAEGGGIPWYTKSGRQEYYMDDPRLIDG
Hydrogenobaculum_sp.          798  L...VMTRFYPKFIGY..............DQTVD..GKPWYTKTGRLEFYREEDTFINV
Beggiatoa_sp.                 971  EDIQRQPRRLLTSPCWSGLMNEGRAYSPFCMSVDR..LVPWRTLTGRQHFYLDHEGYLAF
Thiocapsa_sp._KS1            1002  PDLISQPRRALTSPHWSAIETKGRTYSPWTLNIEA..LKPFHTLTGRQEIYYDHRMFRDL
Nitrolancea_hollandica_Lb     973  FDLTRQVRRTLISPCWTGMVNDGRAYSAWCLNVER..LIPWRTLSGRQHHYMDHPYYIDY
Nitrobacter_winogradskyi      979  YDLTRQPRRILTSPCWTGLVNDGRAYSAWCMNIER..LVPWRTLTGRQSLYLDHQWYLDF
Nitrobacter_hamburgensis      979  YDLTRQPRRLLTSPCWTGMMNDGRAYSAWCMNVER..LVPWRTLTGRQTLYIDHQWYLDF
Nitrococcus_mobilis           984  YDLTRQPRRVLVSPVWTGNITEGRAYAAWCLQVER..LIPWRTLTGRQQMYIDHPWYLDF
Escherichia_coli              998  RDIQAQPRKIISSPTWSGLEDEHVSYNAGYTNVHE..LIPWRTLSGRQQLYQDHQWMRDF
Aromatoleum_aromaticum        781  A...HANEVDVTKPIY........PMRWH..FDD..KKVFPTHTRRAQFYLDHDWYLEA

                  β28           β29                                      α32      β30
Kuenenia_stuttgartiensis
Kuenenia_stuttgartiensis      841  GENFIVHREGPEATPYLPNAIVSTN..PYIR...PDDYGIPE...NAEYWEDRTVRNIKK
Jettenia_caeni                840  GENFIVHREGPEATQYLPNVIVSTN..PYIR...PDDYGIPE...DAEHWDERTVRNIKK
Brocadia_fulgida              840  GENFIVHREGPEATQYLPNVIVSTN..PYIR...PDNYGIPE...SAEHWDERTVRNIKK
Scalindua_brodae              842  GENFVCHREAIEATPYLPNVIVSTN..PYIR...PEDYGIPQ...SHMGADERHIRNIKM
Nitrospira_defluvii           838  GENFISHREGPECTPYMPNAIMTTN..PYVR...PEDYGIPV...TAQHHDDKTVRNIKL
Nitrospira_nitrificans        837  GENFISHREGPEATPYLPNAIFTTN..PYVR...PDDYGIPI...AAQHHDDKMIRNIKL
Nitrospira_inopinata          837  GENFISHREGPEATPYLPNAIFTTN..PYVR...PDDYGIPI...TAQHHDDKTVRNIKL
Nitrospina_gracilis           843  GENFIVHREGPEATPYLPNVIVSTN..PYIR...PDDYGIPE...DEQDPDLRHVRNIKK
Nitrotoga_sp.                 857  GENLTVYRTPIDSSHYEPNVIVGNSRAFALM.ETPEMRGLERMGNSLKIAENRMGRNVIL
Hydrogenobaculum_sp.          839  GENLIVHREAIDSTPYEPNTIVIAKKHPLLRPLDPKSYGLSS.EEMLKDTELRQARAVFI
Beggiatoa_sp.                1029  GEHLPTYKPKADPMLYGDFV..KT....................................
Thiocapsa_sp._KS1            1060  GEDFPVYKPPVDMVSIGDVA..QK....................................
Nitrolancea_hollandica_Lb    1031  GEQLMTHKPKLDPRKTGDIV..RS....................................
Nitrobacter_winogradskyi     1037  GEHIPTYKPRLNPRKTGDIV..KS....................................
Nitrobacter_hamburgensis     1037  GEHIPTYKPRLNPRKTGDIV..KS....................................
Nitrococcus_mobilis          1042  GAQLCTYRAKIDYKDIHDLS..VS....................................
Escherichia_coli             1056  GESLLVYRPPIDTRSVKEVI..GQ....................................
Aromatoleum_aromaticum        825  GESLPTHKDTPM..V.....................................

               α33        α34           β31                   α35
Kuenenia_stuttgartiensis
Kuenenia_stuttgartiensis      893  SWEETKKTKNFLWE.KGYHFYCVTPKSRHTVHSQWAVTDWNFIWNNNFGDPYRMDKRMPG
Jettenia_caeni                892  SWAETKKTKNFLWE.KGYKFYCVTPKSRHTVHSQWAVTDWNFIWNNNFGDPYRMDKRMPG
Brocadia_fulgida              892  SWSDTKQTRNFLWE.KGYKFYCVTPKSRHTVHSQWAVTDWNFIWNNNFGDPYRMDKRMPG
Scalindua_brodae              894  PWSEVKKTKNPLWE.KGYRFYCVTPKTRHRVHSQWSTVDWNLIWNNNFGDPYRMDKRSPG
Nitrospira_defluvii           890  PWSEIKQHPNPLWE.KGYQFYCVTPKTRHRVHSQWSVNDWVQIYESNFGDPYRMDKRTPG
Nitrospira_nitrificans        889  AWQEIKRHSNPLWE.KGYQFYCVTPKTRHRVHSQWSVNDWVQIYESNFGDPYRMDKRTPG
Nitrospira_inopinata          889  SWDEIKRHSNPLWE.KGYQFYCVTPKTRHRVHSQWSVNDWVQIYESNFGDPYRMDKRTPG
Nitrospina_gracilis           895  PWSSVRTTKNFLWE.KGYRFYCVTPKSRHTAHSSWATTDWNMIWNNNFGDPYRMDKRSPG
Nitrotoga_sp.                 916  TTKELMATTHPLRP.HGYEFCFNSPKYRHGAHTTPIDTDLMTLWWGPFGDIYRHDKRQPS
Hydrogenobaculum_sp.          898  SPAKLKDTKHPLRVSFGATHIVHTPKFRHTTHSATGDVDIVALLFGPYGDMYRHDKRTPY
Beggiatoa_sp.                1051  ..........QQGEANSIMLNYLTPHGKWHIHSTYYDNHRMLT...............LS
Thiocapsa_sp._KS1            1082  ..........DIKMKGAKLFRFITPHQKWGIHSMFGDSWQMQN...............MF
Nitrolancea_hollandica_Lb    1053  ..........PV.DDESVVLNYITPHGKWHIHSTYYDNHRMLT...............LS
Nitrobacter_winogradskyi     1059  ..........RV.DDRSLVLNYITPHGKWNIHSTYKDNHRLLM...............LS
Nitrobacter_hamburgensis     1059  ..........RV.DDRSLVLNYITPHGKWNIHSTYKDNHRMLM...............LS
Nitrococcus_mobilis          1064  ..........PI.DDKTLILNYVTPHGKWNIHSTYYDNHRMLT...............LS
Escherichia_coli             1078  ..........KSNGNQEKALNFLTPHQKWGIHSTYSDNLLMLT...............LG
Aromatoleum_aromaticum        838  ...........|..GGDHPFKITGGHPRVSIHSTHLTNSHLSR...............LH

                β32   α36           β33                                β34
Kuenenia_stuttgartiensis
Kuenenia_stuttgartiensis      952  VGEHQIHIHPQAARDLGIEDGDYVYVDANPADRPYEGWKPNDSFYKVSRLMLRAKYNPAY
Jettenia_caeni                951  VGEHQIHMNPEAAKDLGINDGDYVYVDANPADRPYEGWKPSDPFYKVARLMLRCKYNPAY
Brocadia_fulgida              951  VGEHQININPQAAKDLGINDGDYVYVDANPADRPYEGWKPSDPFYKVSRLMLRAKYNPSY
Scalindua_brodae              953  AGEHQLHINPAAAKDLAINDGDYIYVDANPVDRPYEGWKPNDPFYKVARLMVRAKYNPSY
Nitrospira_defluvii           949  VGEHQIHINPQAAKDRGINDGDYCYVDGNPVDRPYRGWKPSDPFYKVARLMIRAKYNPSY
Nitrospira_nitrificans        948  VGEHQVHINPQAAKDRGINDGDYVYIDGNPVDRPYRGWKPSDPYYKVARLMIRAKYNPAY
Nitrospira_inopinata          948  VGEHQLHINPQAAKDRGINDGDYVYVDGNPVDRPYRGWKPSDPYYKVARLMIRAKYNPAY
Nitrospina_gracilis           954  VGEWQVHMNPFLCKDLGINDGDYVYVDANPADRPYMGWKPSDPRYKVARLMLRAKYNPAY
Nitrotoga_sp.                 975  VGEGFVDVNPLDAKRFGIDEGDYIWVDADPADRPYKGWKEGTPEYALARFMVRCRYFPGM
Hydrogenobaculum_sp.          958  VVDAFIEINTHDLQKLGINDGDYVWVDADPQDRPFIGWQNKPEEYEVARLLLRVRGSFST
Beggiatoa_sp.                1086  RGIEPFWINHKDADKIGVIDNDWVEVYND................HGVVVTRAVVSARI
Thiocapsa_sp._KS1            1117  RGGPTVWINDDDARDIGVKDNDWVEIFNE................NGIQVARAVVSHTV
Nitrolancea_hollandica_Lb    1087  RGIEPCWLNDKDAARIGIKDNDWVEVYND................NGVMVTRAAVSARV
Nitrobacter_winogradskyi     1093  RGMDPVWINDRDAEKVGIEDNDWVEVYND................NGVVVTRANVSRRI
Nitrobacter_hamburgensis     1093  RGMDPVWINDRDAEKVGIEDNDWVEVYND................NGVVVTRANVSRRI
Nitrococcus_mobilis          1098  RGMDPVWINDKDAERVGLKDNDWVEVYND................NGVVVTRANVSRRV
Escherichia_coli             1113  RGGPVVWLSEADAKDLGIADNDWIEVFNS................NGALTARAVVSQRV
Aromatoleum_aromaticum        869  RGQPVVHMNSKDAAELGIKDGDMAKLFND................FADCEIMVRTAPNV
```

```
                                     β35        α37
Kuenenia_stuttgartiensis
Kuenenia_stuttgartiensis     1012  PYNCTMMKHSAWISSDKTVQAHETRPDGRALSP.SGYQSSFRYGSQQSITRDWSMPMHQL
Jettenia_caeni               1011  PYGVTMMKHSAWISTERSVKAHETRPDGRALSAGTGYQSSFRYGSQQSITRDWSMPMHQL
Brocadia_fulgida             1011  PYGVTMMKHSAWISTERSVKAHESRPDGRALSAGTGYQSSFRYGSQQSITRDWSMPMHQL
Scalindua_brodae             1013  PYHTTMMKHSTNVATEKSVKAHENRKDGMAMSK.DGYLSNFRYGSQQSITRSWLMPMHQT
Nitrospira_defluvii          1009  PYHVTMAKHAPYVSTAKSVKGHETRPDGRAIAVDTGYQSNFRYGAQQSFTRSWLMPMHQT
Nitrospira_nitrificans       1008  PYHVTMAKHAPYVSTAKSVKGHETRPDGRAIAIDTGYQSNFRYGAQQSFTRSWLMPMHQT
Nitrospira_inopinata         1008  PYHVTMAKHAPFVATPKSVKGHETRPDGRAIAIDTGYQSNFRYGCQQSFTRNWLMPMHQT
Nitrospina_gracilis          1014  PYHTAMMKHSCWTSTERSVKAHEERPDGRALSMTTPYQSNFRYGGQQSITRSWLMPMHQT
Nitrotoga_sp.                1035  SQGSMRMYYNAYAATYGSMEGARTRADGLAKSPRTNYQAMFRSGNHQSCTRAWINPTNTT
Hydrogenobaculum_sp.         1018  PPGLAKIWFNMYGSSHGSVKGTKVNKNGLAENPVTGYQSFYRRGSQQSVTRGWLNPTLMG
Beggiatoa_sp.                1129  PQGVCIWYHSPERTI..SIPKSPLRG.............GRRAGGHNSLTRTRLKPVLMM
Thiocapsa_sp._KS1            1160  PRDMSIVYHQTERHV..NVPFSSLAR........ERGASDLRGGNNNATTRIMMNPATMV
Nitrolancea_hollandica_Lb    1130  QMGTCMIYHAPERTI..SIPKSQVRG.............NRRGGAHNSLTRTRINPMQMA
Nitrobacter_winogradskyi     1136  QPGTCMYYHAVERTV..YIPKSQERK..............WRGGGHNSLTRIRINPLFLA
Nitrobacter_hamburgensis     1136  QPGTCMYYHAVERTV..YIPKSQERK..............WRGGGHNSLTRTRINPLFLA
Nitrococcus_mobilis          1141  QSGMCLYYHAVERTI..YIPKSQIRG.............GRRAGGHNSLTRTRLNPLYLA
Escherichia_coli             1156  PAGMTMMYHAQERIV..NLPGSEITQ..............QRGGIHNSVTRITPKPTHMI
Aromatoleum_aromaticum        912  QPKQCIVYFWDAHQY..........KG..............WK..PYDILLIGMPKPLHLA

                                     β36   β37            β38          α38
Kuenenia_stuttgartiensis
Kuenenia_stuttgartiensis     1071  DSLFHKAKIGMKFIFGFEADNHCINTVP.KETLVKITKAENGGMGGKGVWDPVKTGYTAG
Jettenia_caeni               1071  DSLFHKAKIGMKFVFGFEADNHGINTVP.KETLVKITKAEDGGIGGKGLWDPAKTGYTAG
Brocadia_fulgida             1071  DSLFHKSKTSMKFIFGYEADNHGINTTP.KETLVKITKAEDGGMGGKGVWDPAKTGYTAG
Scalindua_brodae             1072  DTLFHKKKVFMGFMHGGEADNHVVNTVP.KETLVRITKAEDGGLGAKGVWEPARTGFTPA
Nitrospira_defluvii          1069  DSLPGKQANALKFKWGFEIDHHAVNTVP.KECLIRITKAEDGGIGARGPWEPVRTGFTPG
Nitrospira_nitrificans       1068  DSLPGKSANGLKFKWGFEIDHHAVNTVP.KECLIRITKAEDGGIGARGPWEPVRTGFTPG
Nitrospira_inopinata         1068  DSLPGKHAIAWKFKWGYQVDHHAINTVP.KECLIRITKAEDGGIGARGPWEPVRTGFTPG
Nitrospina_gracilis          1074  DSLFHKAKSKMKFMFGYEADNHAINATP.KETLVKFSKAEDGGMHGKGLWEPVRTGYTPE
Nitrotoga_sp.                1095  DTLANKKVFGQEIIIGHQNDVHCANGAP.KESYVKIELAEKGGV.NGGVWHIAAKGYRPT
Hydrogenobaculum_sp.         1078  DTLVRKDLFGQTLNKGFMLDVYCPTGAP.REGFAKITKAENGGIGNVGLWRPLSIGFRPQ
Beggiatoa_sp.                1174  GGYGQ.........FTFHLNYWGPTGVN.RDTHVMVHYSRGVS....GFFLLT.......
Thiocapsa_sp._KS1            1210  GGYAN.........WTYWLNYQGTSPSE.RDCVVLIRKKPMETRGRKVIYQESQL..NVG
Nitrolancea_hollandica_Lb    1175  GGYAQ.........WTYMWNYWGPIGILTRDTHVAVR.......................
Nitrobacter_winogradskyi     1180  GGYAQ.........FTYGWNYWGPTGIFTRDTHVVVRKMEKVE....W............
Nitrobacter_hamburgensis     1180  GGYAQ.........FTYGWNYWGPTGILTRDTHVVVRKMEKLE....W............
Nitrococcus_mobilis          1186  GGSGQ.........FTYAWNYWGPTGIMTRDTHVAVRKLEKLE....W............
Escherichia_coli             1200  GGYAH.........LAYGFNYYGTVGSN.RDEFVVVRKMKNID....WLDGEGND..QVQ
Aromatoleum_aromaticum        947  GGYEQ.........FRYYFMNGSPAPVTDRGVRVSIKKA.....................

                                        α39     β39
Kuenenia_stuttgartiensis
Kuenenia_stuttgartiensis     1130  NE..NDFMKKFLNGELIKVDA.
Jettenia_caeni               1130  NE..NDFMKKYLSGELIKVEKA
Brocadia_fulgida             1130  NE..NDFMKRYLNGELIKIEKA
Scalindua_brodae             1131  NE..SKFMKLYLQGGTTSVE..
Nitrospira_defluvii          1128  QE..NEFMVKWLKGEHIKIKV.
Nitrospira_nitrificans       1127  QE..NEFMIKWLKGEHIKIKV.
Nitrospira_inopinata         1127  QE..NEFMIKWLKGEHIKIKV.
Nitrospina_gracilis          1133  SPLKDRFAEMYLAGQY......
Nitrotoga_sp.                1153  YE..SLQMRTYLKGAFVSR...
Hydrogenobaculum_sp.         1137  NP..NKYFKKYLEAGYVIFV..
Beggiatoa_sp.                      ......................
Thiocapsa_sp._KS1            1258  A.....................
Nitrolancea_hollandica_Lb          ......................
Nitrobacter_winogradskyi           ......................
Nitrobacter_hamburgensis           ......................
Nitrococcus_mobilis                ......................
Escherichia_coli             1244  ESVK..................
Aromatoleum_aromaticum             ......................
```
