# Supplementary figures and images for: Structural and functional characterization of the intracellular filament-forming nitrite oxidoreductase multiprotein complex
Source: Nat Microbiol. 2021 Jul 15;6(9):1129–39. doi: 10.1038/s41564-021-00934-8 (PMC8387239; doi:10.1038/s41564-021-00934-8)

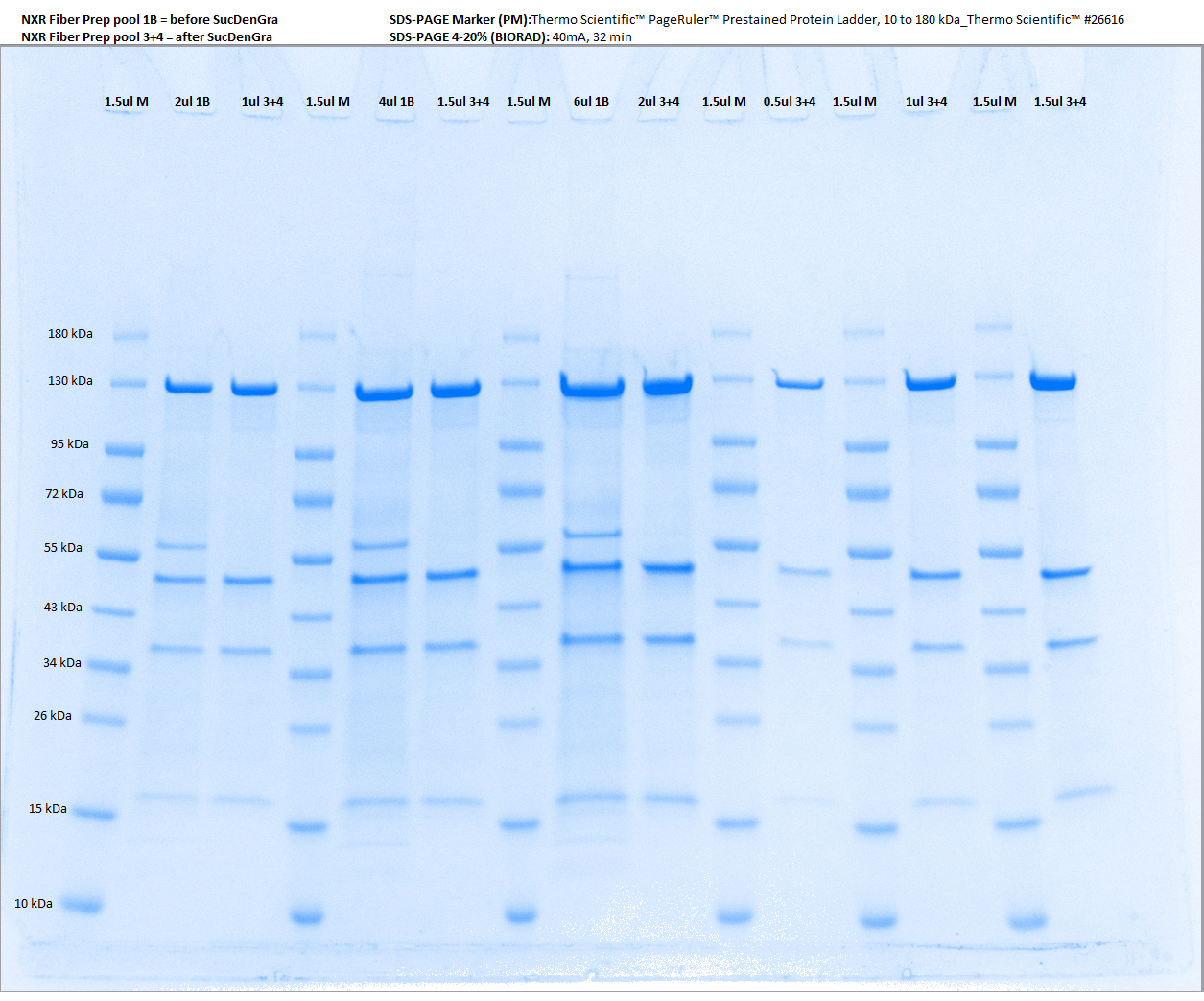

Supplement: Source Data Extended Data Fig. 5c — Unprocessed gel from Extended Data Fig. 5c. [file 41564_2021_934_MOESM6_ESM.png]
